# Synthesis, characterisation, and solution behaviour of Ag(I) *bis*(phenanthroline-oxazine) complexes and the evaluation of their biological activity against the pathogenic yeast *Candida albicans*

**DOI:** 10.1007/s10534-023-00513-w

**Published:** 2023-06-28

**Authors:** Clara Evans, Muhib Ahmed, Darren F. Beirne, Malachy McCann, Kevin Kavanagh, Michael Devereux, Denise Rooney, Frances Heaney

**Affiliations:** 1 Department of Chemistry, Maynooth University, Co. Kildare, Maynooth, Ireland; 2 Kathleen Lonsdale Institute for Human Health Research, Maynooth University, Co. Kildare, Maynooth, Ireland; 3 The Centre for Biomimetic and Therapeutic Research, Focas Research Institute, Technological University Dublin, Dublin 8, Ireland; 4 Department of Biology, Maynooth University, Co. Kildare, Maynooth, Ireland

**Keywords:** Antifungal activity, Culture media, Incubation time, Silver(I) complexes, Phenanthroline, Dynamic solution behaviour

## Abstract

Three Ag(I) *bis*(phenanthroline-oxazine) complexes with varying lipophilicity were synthesised and characterised. The solution stoichiometry of 1:2 Ag(I):ligand was determined for each complex by the continuous variation Job’s plot method using NMR spectroscopy. NMR studies were also carried out to investigate the fluxional behaviour of the Ag(I) complexes in solution. The biological activity of the silver(I) complexes and the corresponding ligands towards a clinical strain of *Candida albicans* MEN was studied using broth microdilution assays. Testing showed the choice of media and the duration of incubation were key determinants of the inhibitory behaviour towards *Candida albicans,* however, the difference between freshly prepared and pre-prepared solutions was insignificant in minimal media. The activity of the metal-free ligands correlated with the length of the alkyl chain. In minimal media, the methyl ester phenanthroline-oxazine ligand was effective only at 60 μM, limiting growth to 67% of the control, while a 60 μM dose of the propyl ester analogue limited fungal growth at < 20% of the control. MIC_50_ and MIC_80_ values for the propyl and hexyl ester analogues were calculated to be 45 and 59 µM (propyl), and 18 and 45 µM (hexyl). Moreover, in a study of activity as a function of time it was observed that the hexyl ester ligand maintained its activity for longer than the methyl and propyl analogues; after 48 h a 60 μM dose held fungal growth at 24% of that of the control. Complexation to Ag(I) was much more effective in enhancing biological activity of the ligands than was increasing the ester chain length. Significantly no difference in activity between the three silver(I) complexes was observed under the experimental conditions. All three complexes were substantially more active than their parent ligands against *Candida albicans* and AgClO_4_ and the three silver(I) *bis*(phen-oxazine) complexes have MIC_80_ values of < 15 μM. The ability of the silver(I) complexes to hold fungal growth at about 20% of the control even after 48 h incubation at low dosages (15 μM) showcases their superiority over the simple silver(I) perchlorate salt, which ceased to be effective at dosages below 60 μM at the extended time point.

**Supplementary Information:**

The online version contains supplementary material available at 10.1007/s10534-023-00513-w.

## Introduction

The antimicrobial activity of silver and its compounds, combined with the low toxicity of silver (Ag(0) and Ag(I)) towards humans, is well established (Lansdown 2006; Möhler et al. 2018) and there are currently a number of commercial silver-based topical treatments, including silver(I) sulfadiazine (Silvadene) which is used to treat burns and a dilute aqueous solution of AgNO_3_ which is used to treat bacterial conjunctivitis in neonates (Kastelan et al. 2018; Shahzad & Ahmed 2013). In addition, Ag(0) and Ag(I) have been used extensively in a range of wound dressings (Boateng & Catanzano 2020). The increased incidence of infections caused by drug-resistant fungi is a serious but often overlooked public health concern. Immunocompromised individuals are a particular concern for opportunistic fungal infections and it is estimated that globally 150 million people are affected by serious fungal infections each year resulting in 1.6 million deaths (Bongomin et al. 2017). There is currently an urgent need to develop novel antifungal agents and there has been a significant interest in creating silver nanoparticles and silver(I) complexes with antimicrobial properties (Medici et al. 2016; Möhler et al. 2018; Savić et al. 2020; Vagabov et al. 2008). The antifungal activity of silver(I) complexes is linked to their ability to dissociate within the cell and release Ag(I) ions which can bind to proteins in fungal cell walls leading to a rapid efflux of potassium ions from the cell (Vagabov et al. 2008). In addition, the Ag(I) ions cause changes in the morphology of yeast mitochondria (Yang & Pon 2003) and they bind to intracellular enzymes, inhibiting their functions and resulting in cell death (Hecel et al. 2019).

**Scheme 1:**
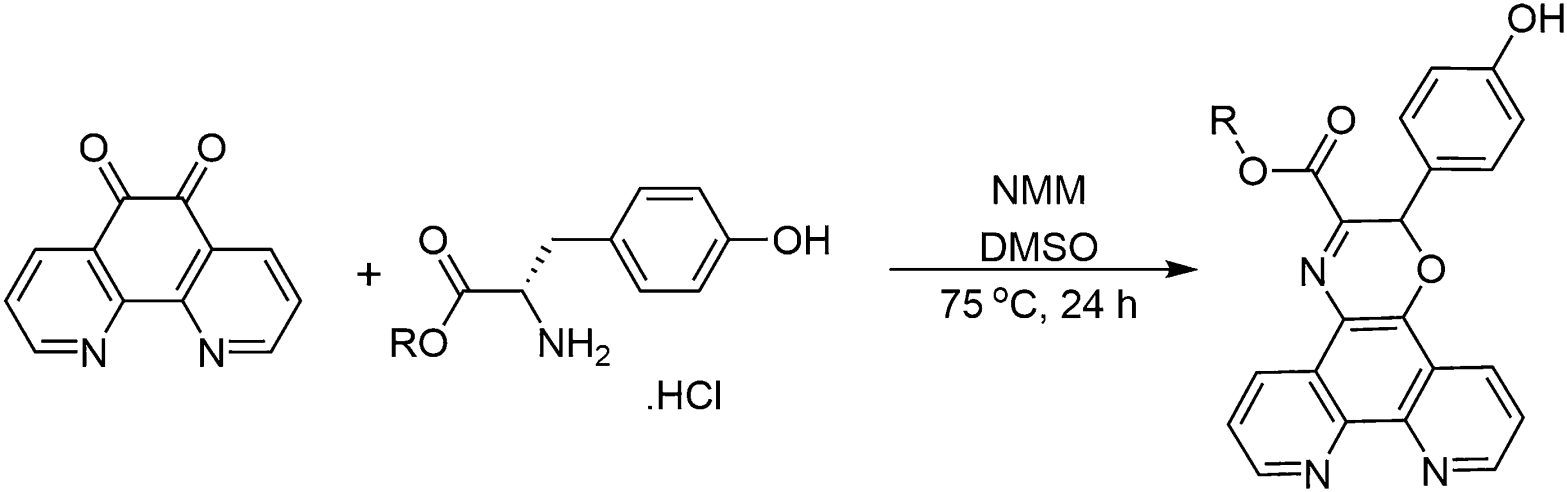
Reaction of 1,10-phenanthroline-5,6-dione with amino acid ester hydrochlorides of L-tyrosine to form phen-oxazines **1**, **2** and **3**, [NMM = *N*-methylmorpholine]

1,10-Phenanthroline (phen) has attracted much interest in the field of cell biology as its rigid planar structure makes it a suitable DNA intercalator, while the juxtaposition of the two nitrogen atoms on the fused aromatic rings allow it to chelate metal ions. There is current interest in the medicinal chemistry of silver(I) complexes containing phen or one of its analogues as ligands, and complexes have been reported which have antibacterial (Ahmed et al. 2019; O'Shaughnessy et al. 2020; Viganor et al. 2017), antifungal (Gandra et al. 2020) and anticancer activity (Aslam et al. 2016; Thornton et al. 2016). Previously, our group synthesised the phenanthroline-oxazine (phen-oxazine) ligand **1** by a reaction of 1,10-phenanthroline-5,6-dione (phendione) with the methyl ester of L-tyrosine (Scheme 1). The Ag(I) and Cu(II) *bis*-complexes of **1** showed stronger binding to calf-thymus DNA than their phen analogues or the minor groove binding drugs, pentamidine and netropsin (McCann et al. 2013). We previously carried out a study focused on the antibacterial (*Escherichia coli, Staphylococcus aureus* and methicillin-resistant *S. aureus *(MRSA)) properties of Cu(II) complexes of **1** and a series of more lipophilic analogues (Ahmed et al. 2022). These studies showed that the lipophilicity of the ligand did influence the antibacterial activity, with the complexes containing the hexyl and octyl alkyl chains showing the highest activity. Herein, we extend this study to investigate the antifungal properties of the Ag(I) *bis*-complex of **1** and its propyl **2** and hexyl **3** analogues (Scheme 1).

The labile nature of the Ag–N bond in silver(I) *bis*(phenanthroline) complexes has been exploited in catalysis and in the synthesis of metal phenanthroline complexes (Huang et al. 2017; Kharmawphlang et al. 1995). It is well-known that catalyst design is challenged due to the fluxional behaviour of the complexes, which can alter the nuclearity of the complex in solution (Huang et al. 2017). This accords with our previous study on a *bis*(phenanthroline-isoniazid) silver(I) nitrate complex which formed a polymeric crystalline material upon slow diffusion of DCM into a solution of the complex in DMSO (Ahmed et al. 2019). Clearly, the speciation of a complex in solution is important in understanding its mode of activity, and recently there have been a number of interesting studies probing this effect with phenanthroline complexes. The first of these investigated the stability of some Cu(II) *bis*(phenanthroline) complexes in cell growth medium and related it to the cytotoxicity of the complexes towards the A2780 cell line (Nunes et al. 2020). In a second publication a real-time electrochemical study on the extracellular speciation of *bis*(1,10-phenanthroline)Ag(I) acetate in the presence of A549 lung cancer cells was reported (Sidambaram and Colleran 2021). Both of these studies showed that ligand dissociation does occur. Therefore, as part of our current study we set out to investigate the dynamic behaviour of Ag(I) *bis*(phen-oxazine) complexes in solution. We also set out to investigate if the complexes were stable in the fungal growth media and to see the influence of media on their level of activity against *C. albicans*.

## Materials and methods

All chemicals were reagent grade and used without further purification, unless stated otherwise. 1,10-Phenanthroline-5,6-dione (phendione) was synthesised by a literature procedure (Zheng et al. 2010). Caution: extreme care must be exercised when handling perchlorate salts. Nutrient broth and Phosphate Buffered Saline (PBS) were obtained from Sigma Aldrich and prepared according to the manufacturer’s instructions. NMR spectra were recorded on a Bruker Avance spectrometer operating at 500 MHz for the ^1^H nucleus and 126 MHz for the ^13^C nucleus. The probe temperature was maintained at 25 °C. Residual solvent peaks were used as internal standard. FTIR spectra were recorded on a Perkin Elmer Spectrum 100 FT-IR spectrometer or a Nicolet iS50 FT-IR instrument. High Resolution Mass Spectrometry (HRMS) analysis was carried out on a Bruker MaXis HD ESI-QTOF mass spectrometer. CHN elemental analysis was carried out on a FLASH EA 1112 Series Elemental Analyser with Eager 300 operating software.

To determine the solution stoichiometry of the Ag(I):ligand complex, a Job’s Plot was created for each complex (Gil and Oliveira 1990). This was done by making a series of solutions of AgClO_4_:ligand with a total concentration of 40 mM and recording the ^1^H NMR spectrum for each sample. A set of proton signals from each spectrum was compared with the corresponding signals of the free ligand and the difference in ppm (∆δ) was recorded. A plot was made of mole fraction of ligand (X_L_) against ∆δ multiplied by X_L_ and the stoichiometry evaluated through the maximum of the bell-shaped curves. The solutions were held at 25 °C.

The growth inhibition ability of compounds **1–6**, and AgClO_4_ towards *Candida albicans* was determined using the broth microdilution susceptibility protocol method (Berkow et al. 2020). Compounds were serially diluted on 96-well plates starting at a concentration of 60 µM; all experiments were repeated in triplicate.

### Statistical analysis

Two-way ANOVA (ANalysis Of VAriation) data sets were generated to determine the statistical significance of the data associated with inhibition of *C. albicans* growth by, and between, the test compounds **1**–**6**, AgClO_4_ and the control experiments. Analysis was performed using GraphPad Prism v10 Software. Statistically significant results were considered as those with *P* ≤ 0.05; where *P* > 0.05 results were interpreted to be non-significant (Rodríguez-Arias et al. 2022).

### Synthesis of compounds

Ligands **1–3** and silver(I) complex [Ag(**1**)_2_](ClO_4_) (**4**) were synthesised according to literature procedures (Ahmed et al. 2022; McCann et al. 2013). All silver(I) complexes were stored in the dark.

[Ag(**2**)_2_](ClO_4_) (**5**).

A 10 mL ACN solution of AgClO_4_ (0.054 g, 0.25 mmol) was added to 90 mL of heated ACN containing **2** (0.52 mmol). The solution was heated at reflux for 2 h in the absence of light. The resulting yellow solution was allowed to cool to room temperature and then reduced *in vacuo* to ~ 5 mL. The product was precipitated by addition to 400 mL of cold diethyl ether. The product was retrieved from the mixture via vacuum filtration, washed with three portions of 50 mL cold diethyl ether and dried under vacuum.

Yellow solid. **Yield:** 0.191 g, 75%. ^**1**^**H NMR** (DMSO-d6): **δ** 9.78 (br s, 1H, O**H**), 9.12 (d, *J* = 3.1 Hz, 1H, phen**H**), 9.05 (d, *J* = 3.2 Hz, 1H phen**H**), 9.02 (d, *J* = 8.2 Hz, 1H, phen**H**), 8.75 (d, *J* = 7.9 Hz, 1H, phen**H**), 8.01 (dd, *J* = 4.4, 7.9 Hz, 1H, phen**H**), 7.96 (dd, *J* = 4.4, 8.0 Hz, 1H, phen**H**), 7.24 (d, *J* = 8.4 Hz, 2H, Ar**H**), 6.70 (d, *J* = 8.5 Hz, 2H, Ar**H**), 6.64 (s, 1H, oxazineC**H**), 4.33 – 4.24 (m, 2H, -OC**H**_**2**_), 1.74 – 1.70 (m, 2H, C**H**_**2**_), 0.93 (t, *J* = 7.3 Hz, 3H, C**H**_**3**_). ^**13**^**C NMR** (DMSO-d6): **δ** 161.9 (**C** = O), 158.9 (**C**–OH), 152.6 (phen**C**-H), 151.1 (oxazine-**C** = N), 149.9 (phen**C**-H), 142.8 (phen**C**), 138.7 (oxazine-**C**O), 138.7 (Ar**C**), 133.0 (phen**C**-H), 131.9 (phen**C**-H), 129.2 (Ar**C**-H), 126.8 (oxazine-**C**N), 125.7 (phen**C**-H), 125.4 (phen**C**-H), 124.8 (phen**C**), 122.0 (phen**C**), 121.7 (phen**C**), 115.9 (Ar**C**-H), 72.6 (oxazine**C-**H), 67.4 (O**C**H_2_), 21.5 (**C**H_2_), 10.2 (**C**H_3_). **FTIR** (ATR, cm^−1^): 3356, 1737, 1601, 1508, 1437, 1220, 1097, 963, 808, 734. **UV/vis** (390 nm. ε = 15,629 M^−1^ cm^−1^). **HRMS** (ESI +); Calcd *m/z* for [Ag(C_24_H_19_N_3_O_4_)_2_]^+^: (M)^+^ 933.1797 Found (M)^+^ 933.1868. **CHN (%):** Calcd: [Ag(C_24_H_19_N_3_O_4_)_2_](ClO_4_).DCM: C 52.59, H 3.60, N 7.50; Found: C 52.15, H 3.64, N 7.39.

[Ag(**3**)_2_](ClO_4_) (**6**) was synthesised using the same procedure to **5** except** 3** was used as the ligand. Yellow solid. **Yield:** 0.148 g, 63%. ^**1**^**H NMR** (DMSO-d6, 500 MHz): δ 9.73 (s, 1H, O**H**), 9.13 (dd, *J* = 1.6, 4.5 Hz, 1H, phen**H**), 9.05 (dd, *J* = 1.5, 4.4 Hz, 1H, phen**H**), 9.0 (dd, *J* = 1.6, 8.3 Hz 1H, phen**H**), 8.76 (dd, *J* = 1.6, 8.3 Hz, 1H, phen**H**), 8.02 (dd, *J* = 4.5, 8.3 Hz, 1H, phen**H**), 7.95 (dd, *J* = 4.5, 8.3 Hz, 1H, phen**H**), 7.23 (d, *J* = 8.7 Hz 2H, Ar**H**), 6.68 (d, *J* = 8.7 Hz, 2H, Ar**H**), 6.63 (s, 1H, oxazine-C**H**), 4.35—4.25 (m, 2H, -OC**H**_2_), 1.68—1.64 (m, 2H, C**H**_2_), 1.29—1.27 (m, 6H, 3 × C**H**_2_), 0.86 (t,* J* = 6.8 Hz 3H, C**H**_3_). ^**13**^**C NMR** (DMSO-d6, 126 MHz): **δ** 161.8 (**C** = O), 158.9 (**C**–OH), 152.5 (phen**C**-H), 151.1 (oxazine-**C** = N), 149.9 (phen**C**-H), 142.7 (phen**C**), 138.7 (oxazine-**C**O), 138.3 (Ar**C**), 133.0 (phen**C**-H), 131.9 (phen**C**-H), 129.1 (Ar**C**-H), 126.8 (oxazine-**C**N), 125.7 (phen**C**-H), 125.3 (phen**C**-H), 124.7 (phen**C**), 121.9 (phen**C**), 121.6 (phen**C**), 115.8 (Ar**C**-H), 72.7 (oxazine-**C**H**),** 65.9 (O**C**H_2_), 30.8 (OCH_2_**C**H_2_), 27.9 (**C**H_2_), 24.9 (**C**H_2_), 22.0 (**C**H_2_), 13.9 (**C**H_3_). **FTIR** (ATR, cm^−1^): 3386, 1735, 1611, 1514, 1436, 1380, 1222, 1096, 807, 734. **UV/vis** (391 nm. ε = 15,877 M^−1^ cm^−1^) **HRMS** (ESI +); Calcd *m/z* for [Ag(C_27_H_25_N_3_O_4_)_2_]^+^: (M)^+^ 1017.2736; Found (M)^+^ 1017.3406. **CHN (%):** Calcd: [Ag(C_27_H_25_N_3_O_4_)_2_](ClO_4_).DCM: C 54.90, H 4.36, N 6.98; Found: C 54.54, H 4.19, N 6.45.

### General procedures for biological testing

Minimal media was prepared by dissolving D-glucose (2% w/v), yeast nitrogen base (0.17% w/v) and ammonium sulfate (0.5% w/v) in deionised water. YEPD media was prepared by dissolving D-glucose (2% w/v), bactopeptone (2% w/v) and yeast extract (1% w/v) in deionised water. Both media were sterilised in an autoclave at 121 °C for 15 min and allowed to cool.

*C. albicans* MEN (serotype B, wild-type originally isolated from ocular infection) was used for all experiments. *C. albicans* was cultured as described in the literature (Brennan et al. 2002). Briefly, *C. albicans* was grown to stationary phase by inoculating 50 mL of the chosen media in a 100 mL conical flask and incubating overnight at 37 °C and 200 rpm in an orbital incubator. The cell density was counted on a Neubauer haemocytometer under a light microscope and found to contain approximately 5 × 10^8^ cells per mL. For each experiment this culture was diluted with the media of choice, (either YEPD or minimal media) to give a cell density of 5 × 10^6^ cells per mL for the *C. albicans* working solution.

### Determination of inhibition of fungal cell growth after 24 h in minimal/nutrient rich media

Stock solutions of compounds **1–6** and AgClO_4_ were prepared at 120 μM in 5% v/v DMSO in media (either YEPD or minimal media) and their ability to inhibit the growth of *Candida albicans* tested according to the CLSI broth microdilution guidelines. Plates were read after 24 h incubation at 37 °C, at 600 nm on a Synergy HT Bio-Tek plate reader. Cells grown in media (minimal media or YEPD) in the absence of any other compound was taken as the control experiment and assigned as having 100% growth (optical density of 1). For each medium (minimal or YEPD) the impact of DMSO (at 2.5% v/v, the highest level in any of the test wells), and of each test compound (60–15 µM) on cell growth was determined as a percentage of the optical density of the respective test wells relative to that of the control. Experiments were repeated in triplicate and results taken as an average of the three readings.

### Determination of the initiation and duration of inhibition of fungal cell growth

The above experiment was repeated (in both minimal and YEPD media) and the plates read at 3, 6, 24 and 48 h after incubation.

### Determination of the impact of pre-preparation of test solutions on inhibition of fungal cell growth

The above experiment was repeated with pre-prepared test solutions. Freshly prepared solutions of each test compound, 120 μM in DMSO 5% v/v in both YEPD and minimal media, were placed in the dark and allowed to stand for 0, 3, 6, 24, 48 and 72 h. After allowing the test solution to stand for the stated time the inhibition of growth experiment was conducted, and plates read after 24 h incubation as described above.

## Results and discussion

### Chemical synthesis

**Fig. 1:**
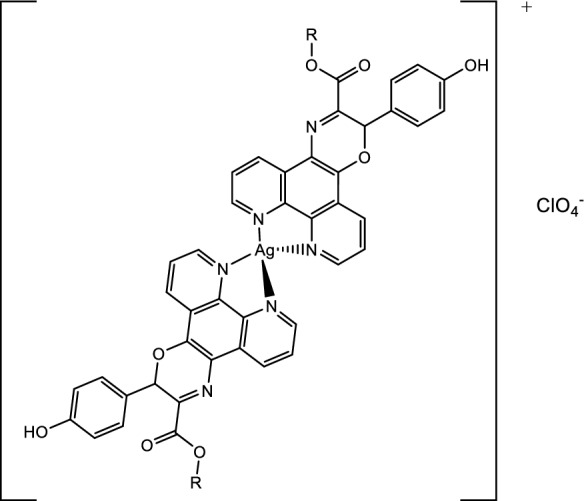
Proposed general structure of silver(I) complexes **4** (R = Me) [Ag(**1**)_2_]ClO_4_, **5** (R = Pr) [Ag(**2**)_2_]ClO_4_ and** 6** (R = Hex) [Ag(**3**)_2_]ClO_4_

The phen-oxazine ligands (**1** R = Me, **2** R = Pr, **3** R = Hex) were synthesised by reacting phendione with the appropriate L-tyrosine ester hydrochloride (Scheme 1) and the compounds were isolated in moderate yield (31–45%) using our previously reported procedure (Ahmed et al. 2022). The silver(I) complexes (**4**–**6**) were synthesised by reacting AgClO_4_ in a 1:2 molar ratio with the ligand and were isolated in 63–75% yield. The complexes were characterised using NMR, and IR spectroscopy, and both high resolution mass spectrometry and elemental analysis supported the Ag(I):ligand formulation as 1:2. The characterisation data for the novel complexes** 5** and **6** are given in **Figures S4**–**S11**, and the proposed solid state general structure for **4**–**6** is shown in Fig. 1. In keeping with literature precedent for silver complexes, the phenanthroline protons of **1–3** were only slightly deshielded upon complexation to silver; ∆(^1^H)_coord_ Phen-H ~ 0.01–0.18 ppm (DMSO-d6) (Kalinowska-Lis et al. 2015; Savić et al. 2018). The impact of complexation on ^13^C NMR resonances was also very marginal. The limited effect of silver complexation on the chemical shifts of **1–3** is compatible with fast ligand exchange for **4–6** in solution on the NMR time scale. Intense absorptions in the IR spectra between 1000 and 1100 cm^−1^ is in keeping with a free, uncomplexed, perchlorate anion (Lewis et al. 1975; Schilt and Taylor 1959). Further, as previously observed for their copper analogues (Ahmed et al. 2022) complexation to the Ag(I) centre causes the absorption associated with out of plane bending of the phen C-H bonds to shift to slightly lower wavenumber, *e.g.* from 739 to 733 cm^−1^ for the ligand **1** complex **4** pair.

### Stoichiometry of the silver(I) complexes in solution

**Fig. 2:**
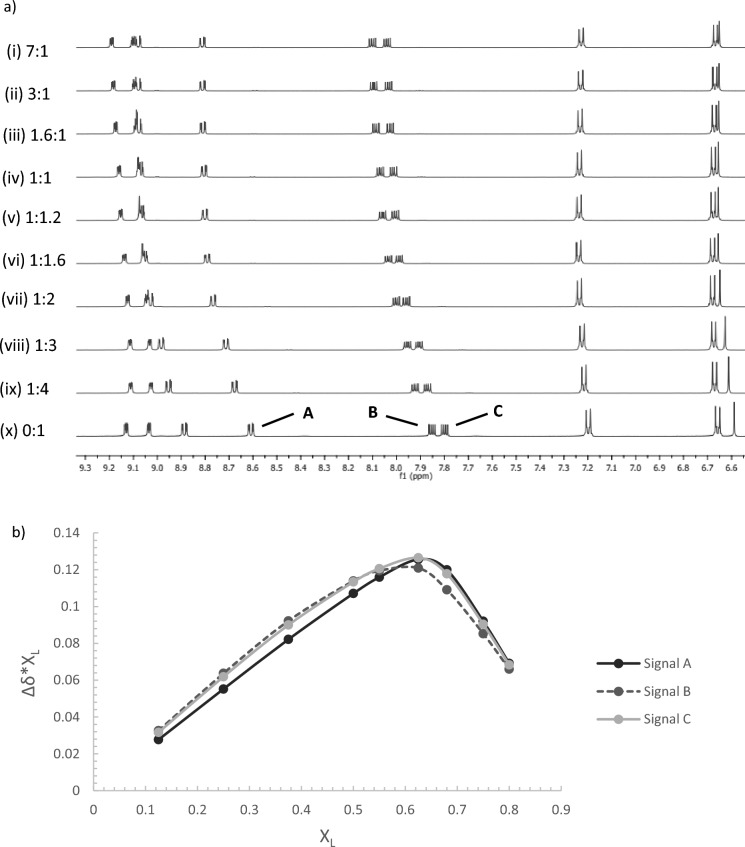
**a** ^1^H NMR spectra of solutions of AgClO_4_ and **1** with a fixed total concentration of 40 mM in DMSO-d6 at 25 °C, with relative ratios varying from (i) 7:1 (AgClO_4_:**1**) to (x) 0:1 (AgClO_4_:**1**). **b** Job’s Plot of various AgClO_4_:**1** solutions in DMSO-d6 where X_L_ is the mole fraction of** 1**, Δδ*X_L_ is the difference in chemical shift (with reference to free **1** signals at 8.60 **A**, 7.85 **B** and 7.79 **C** ppm) multiplied by the mole fraction of **1**

The stoichiometry between Ag(I) and each of the ligands **1**–**3** was evaluated using the continuous variation method (Gil & Oliveira 1990). Figure 2a shows the ^1^H NMR data obtained for **1** upon varying the ratio between AgClO_4_ and **1** with a fixed total concentration of 40 mM in DMSO-d6 at 25 °C. Figure 2b shows the bell-shaped curves constructed from the data using the change that occurs in the chemical shift of the signals for the ‘free’ ligand **1** at 8.60, 7.85 and 7.79 ppm upon addition of the AgClO_4_. It is evident from Fig. 2b that the maximum of each curve is centred close to 0.67, indicating the formation of a 1:2 complex. Similar results were observed when the experiment was repeated with ligands **2** and **3 (Figure S14–S17)**.

### Stability and dynamic behaviour of complexes 4–6 in solution

**Fig. 3:**
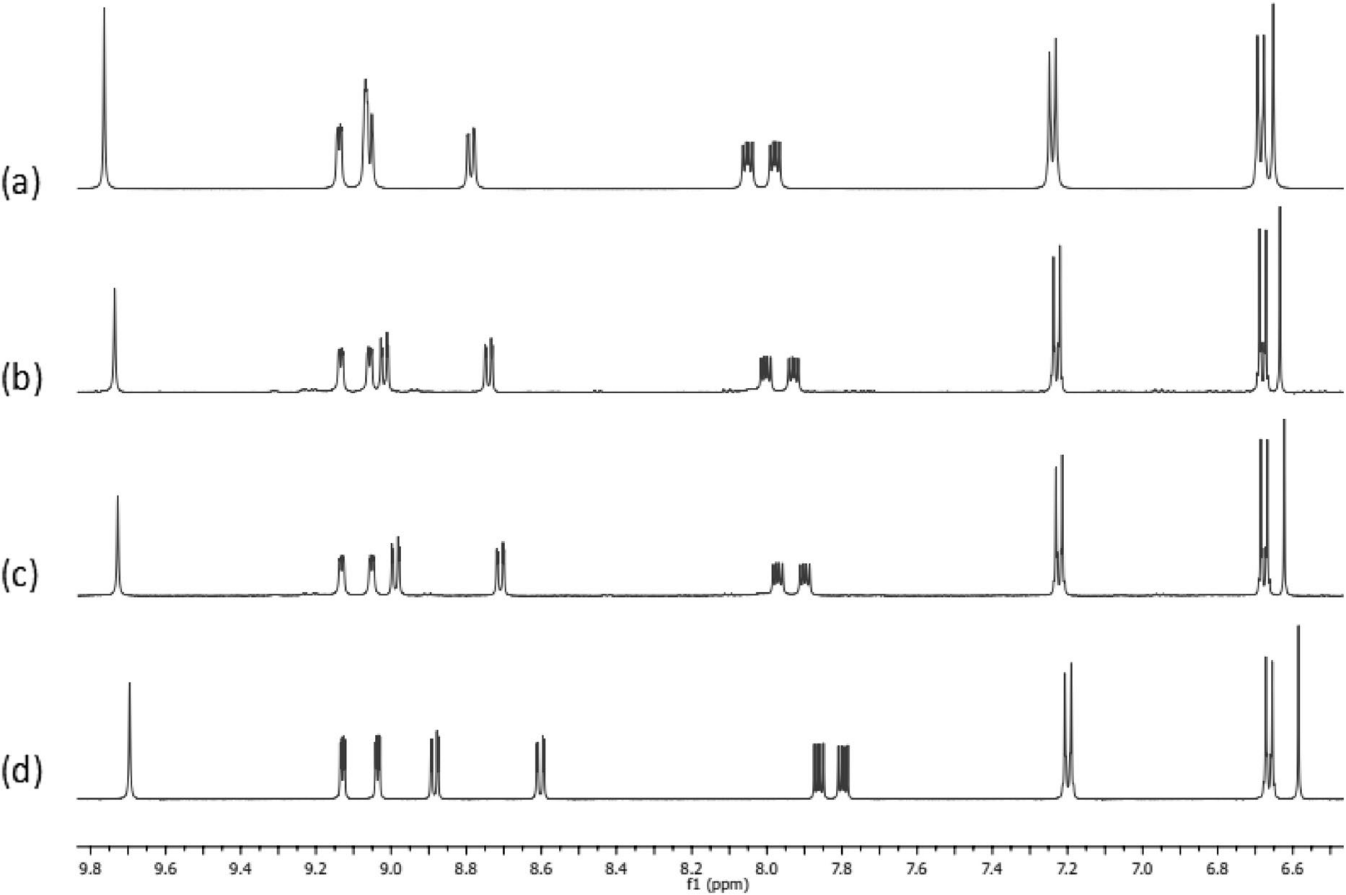
^1^H NMR spectra of **a** complex **5**, **b** ligand **2** and complex **5** in a 1:1 ratio, **c** ligand **2** and complex** 5** in a 2:1 ratio, **d** ligand **2** in DMSO-d6 at 25 °C. Samples were prepared by mixing the required volumes of 10 mM stock solutions of each sample

**Fig. 4:**
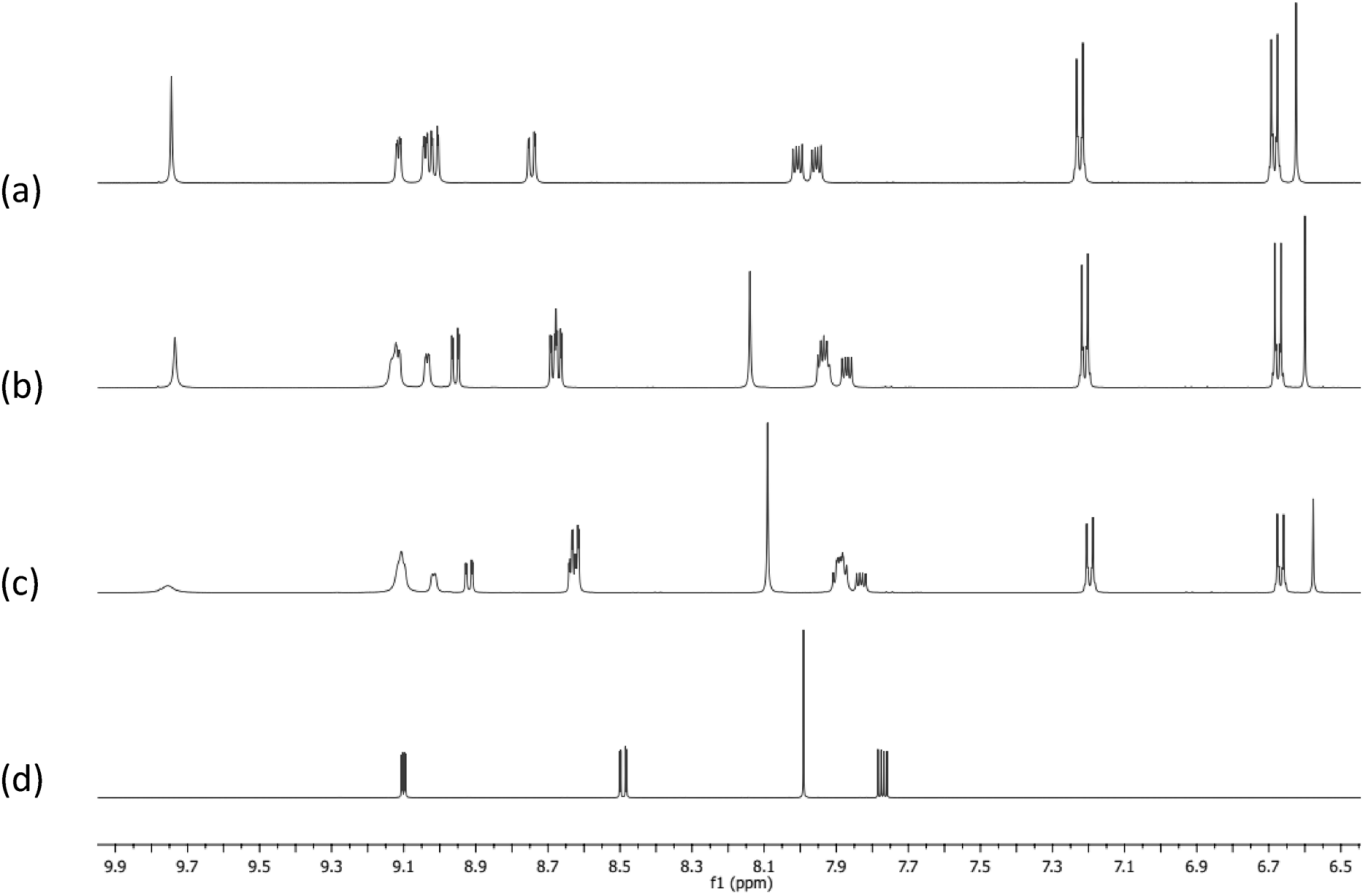
^1^H NMR spectra of **a** complex **6**, **b** phenanthroline and complex **6** in a 1:1 ratio, **c** phenanthroline and complex **6** in a 2:1 ratio, **d** phenanthroline in d6-DMSO at 25 °C. Samples were prepared by mixing the required volumes of 10 mM stock solutions of each sample

The *d*^10^ Ag(I) ion has zero ligand field stabilisation energy and it is known that the Ag–N bonds in Ag(I) complexes containing phenanthroline-like ligands can be highly labile (Huang et al. 2017). With this in mind, the dynamic behaviour of the complexes **4–6** was investigated using ^1^H NMR spectroscopy. ^1^H NMR spectra of the three Ag(I) complexes were recorded in the presence of additional ligand (1:1 and 1:2 ratio), and compared to the spectra of ligand only, and complex only. Figure 3 show the results for this experiment for ligand **2** and complex **5**. Comparing the spectrum of the free ligand (d) with the spectrum of the complex (a), it can be seen that the proton signals in the phenanthroline backbone shift upon complexation to the Ag(I) centre. For example, the doublet signal at 8.59 ppm of **2** is shifted to 8.75 ppm, while the doublet of doublets at 7.85 ppm is shifted to 8.01 ppm and similarly the doublet of doublet signal shifted from 7.79 ppm to 7.96 ppm. Clearly, only one set of signals is observed in the spectra of the complex in the presence of added ligand (Fig. 4(b) and (c), (complex **5**: ligand **2** ratio of 1:1 and 1:2 respectively). This can be rationalized as being due to dissociation and reassociation of the phen-oxazine ligand to the Ag(I) centre at a rate which is faster than the NMR timescale, so an average set of signals are observed. This dynamic behaviour of the Ag(I) *bis*(phen-oxazine) complexes has implications for the biological activity of the complexes as there could be rapid exchange of one or both of the phen-oxazine ligands with ligands that are naturally present in the biological growth medium (*e.g*. water, halide anions, proteins).

**Fig. 5:**
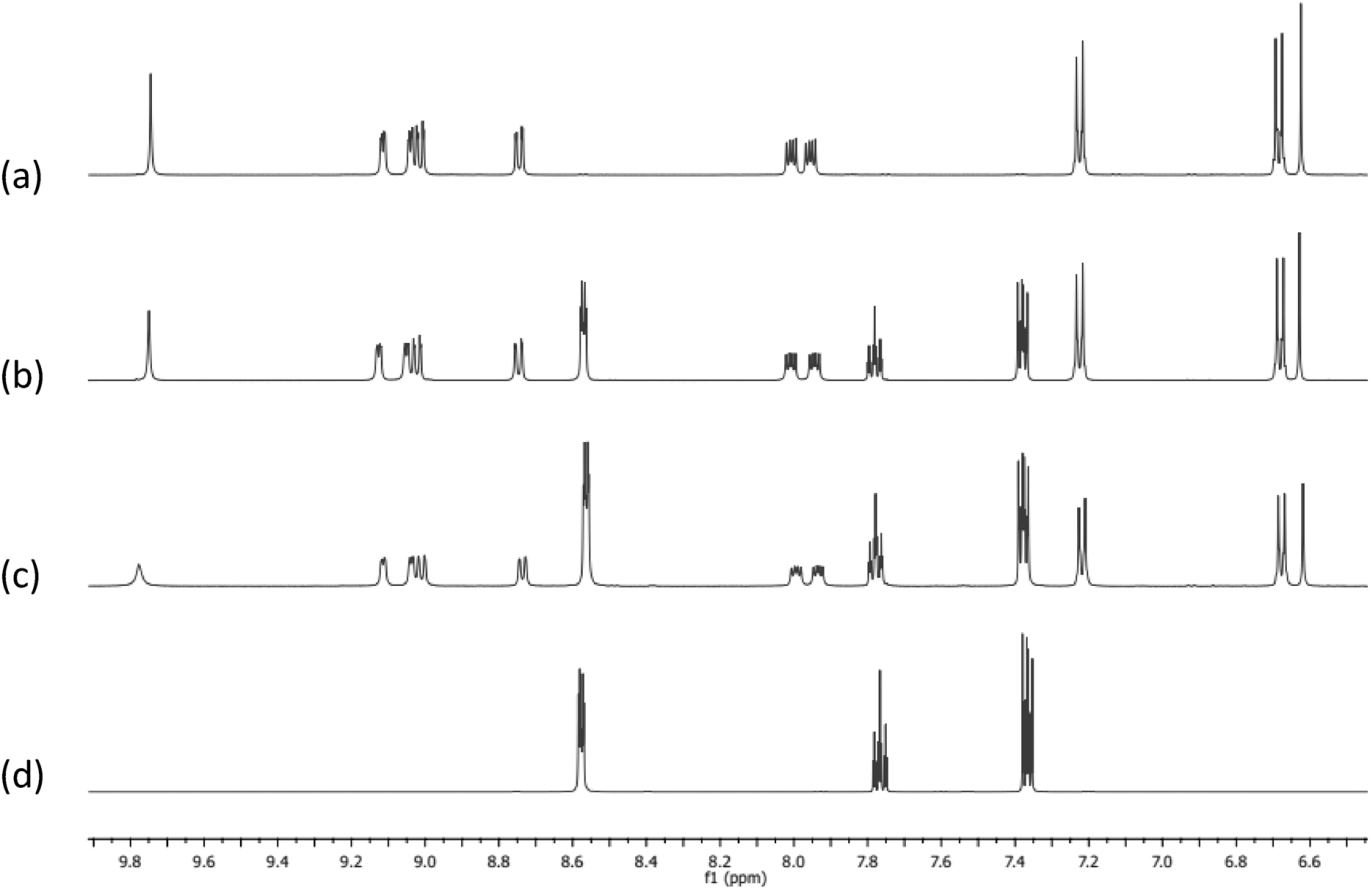
^1^H NMR spectra of **a** complex **6**, **b** pyridine and complex **6** in a 1:1 ratio, **c** pyridine and complex **6** in a 2:1 ratio, **d** pyridine in d6-DMSO at 25 °C. Samples were prepared by mixing the required volumes of 10 mM stock solutions of each sample

When the above experiment was repeated for the complexes with added phenanthroline instead of the phen-oxazine ligands, again an average set of signals was observed. The results of this experiment using complex **6** are shown in Fig. 4. In spectra (b) and (c) in which phenanthroline is added to **6**, the signals for the ‘free’ phenanthroline ligand (spectrum d) and **6** (spectrum a) shifted in position but only one set of signals can be assigned to the phenanthroline and the complex, indicating fast exchange between the chelating phen-oxazine ligands of **6** and the phenanthroline present in solution. When the experiment was repeated using the monodentate ligand, pyridine, and the phen-oxazine complex **6**, no shifts in the signals were observed from the spectra recorded of **6** and of pyridine to that of the mixtures containing complex **6** in the presence of added pyridine. This indicate that pyridine did not compete with the dissociated ligand **3** for complexation to the Ag(I) centre, an observation that is consistent with the chelate effect (Fig. 5).

Recent studies by Colleran and co-workers (Sidambaram and Colleran 2021) of Ag(I)(phenanthroline)_2_ acetate using real-time electrochemistry indicated, in contrast to our findings for DMSO solutions of the present complexes, that ligand dissociation did not occur in acellular media, but when the complex was incubated in the presence of A549 lung cells there was clear evidence for ligand dissociation. The authors proposed that this happened because of the release of reducing agents (*e.g*. ascorbic acid, glutathione or thiol membrane proteins) from the A549 cells into the extracellular matrix. It may be that real-time electrochemistry does not detect fast exchange processes in the manner that NMR spectroscopy can, or that these processes do not occur in biological media compared to an organic solvent like DMSO.

UV–vis absorption spectra were recorded of compounds **1–6** in DMSO (**Figure S18-S23**). The ligands showed intense bands in the visible region 300–450 nm with the lowest energy transition occurring at *ca.* 390 nm (**Table S1**). We propose that this band arises from a π→π* transition. The spectra of the silver(I) complexes **4**–**6** were nearly identical to their respective ligands except that the extinction coefficients associated with the band have approximately doubled in magnitude reflecting the 2:1 ligand:Ag(I) ion stoichiometry of the complexes. So again, we suggest that the lowest energy bands are intraligand bands and arise from π→π* transitions. This assignment is in line with that proposed by other researchers for similar systems (Shahabadi et al. 2011).

To investigate the stability of **4**–**6** in the fungal growth media, a series of investigations were carried out using UV/vis spectroscopy. The UV–vis spectra of solutions of complexes **4–6** in 5% v/v DMSO in minimal media (60 μM) used for the biological testing were recorded over a 72 h time-period (**Figure S24-26**). Complexes **4** and **5** showed some moderate changes in their spectra over that time period, while complex **6** exhibited more significant changes. Over the period of 0–6 h, complex **6** exhibited a decrease in the intensity of the band at 392 nm (λ_max_), and this was followed by a shift of the band to longer wavelength with λ_max_ moving to 401 nm after 24 h. There was also a drift upwards in the position of the baseline. These changes are consistent with aggregation with time of the more lipophilic complex **6** in the polar minimal media (Prabhu et al. 2012).

### In vitro activity against *Candida albicans*

We determined the antifungal potential of the phen-oxazine ligands **1–3** and their silver(I) complexes **4–6** and compared their behaviour to that of silver(I) perchlorate. Standard antimicrobial protocols were employed, which involved incubating the organism with the test compound for 24 h at 37 °C in a cell growing medium held in a 96-well plate (Berkow et al. 2020). With tentative evidence for aggregation of the hexyl complex **6** in biological media and with cognizance that the dynamic solution behaviour observed for the silver(I) complexes **4–6** in DMSO may also influence biological activity it was important to investigate the impact of the growth medium composition and the time spent standing in solution on the activity of each compound, and to explore their duration of effectiveness.

Initial studies, following the most typical protocol, involved 24 h incubation of *C. albicans* with freshly prepared solutions of silver(I) perchlorate, the free ligands and the silver(I) complexes (15–60 μM) in the chosen media. The optical density of each well was measured. The effectiveness of an individual test compound at inhibiting fungal growth was determined as a percentage of the optical density of the test wells with respect to that of the control. Cells were also grown in media with DMSO (2.5% v/v) to explore the potential influence of the organic solvent, this concentration represents the highest level of the organic solvent in any test well. In all cases growth inhibition values were taken as the average of triplicate measurements.

**Fig. 6:**
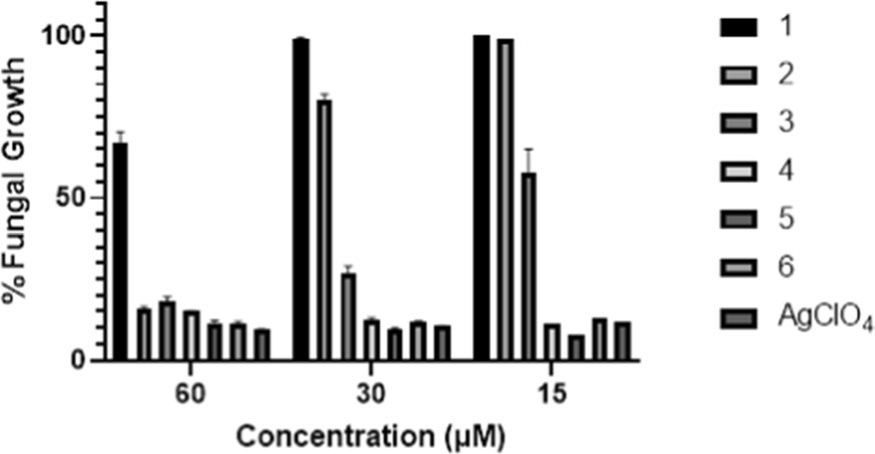
Antifungal activity of test compounds **1**–**6** and AgClO_4_ across the range 60–15 µM measured as their ability to restrict *C. albicans* growth in minimal media after 24 h of incubation

Fungal growth in minimal media with 2.5% v/v DMSO was not significantly different from the control (> 96%), however, all of the test compounds had some impact in this medium (Tables 1** and S2, **Fig. 6**)**. The methyl ester ligand **1** was effective only at the highest dose (60 μM) where growth was limited to 67% of the control. As the hydrocarbon tail lengthened so too did potency; 60 μM doses of either the propyl or hexyl ester substituted ligands **2** or **3** curtailed fungal growth at < 20% of the control. At lower concentrations differences in potency emerged between the ligands; at 30 μM **1** was completely ineffective and **2** was significantly less effective than its hexyl analogue **3** (100%, 80% and 27% fungal growth with respect to the control; *P* < 0.0001 between all pairs of test ligands at 30 μM). At the lowest dose, 15 μM, the only active ligand was **3** with the hexyl ester substitutent. Across the dosage range studied, ligand **1** was too inactive to determine an accurate MIC_50_ value (it is clearly > 60 μM). The lipophilic effect, which may be correlated with permeability through biological membranes, is likely to be the controlling factor for the observed trend in antifungal activity of this series of ligands, with potency increasing between the methyl and hexyl analogues, **1** and **3** (*P* < 0.0001 between test compounds **1** and **3** at all dosages).

**Table 1:** In vitro inhibitory effects of test samples determined as their ability after 24 h incubation to limit the growth of *C. albicans* in minimal media*

Test sample and dosage				Test sample and dosage			
Ligand	60 μM	30 μM	15 μM	Silver(I) Salt	60 μM	30 μM	15 μM
	Percentage growth^#^				Percentage growth^#^		
1	67%	99%	100%	4	15%	12%	11%
2	17%	80%	100%	5	12%	10%	9%
3	18%	27%	57%	6	11%	12%	14%
				AgClO_4_	9%	10%	12%

*When the same experiments were conducted in nutrient-rich YEPD media no inhibition was observed with any test compound across the dosage range studied, 60–15 μM (Table S3 row A)

^#^Percentage Growth is measured relative to a control sample where the *C. albicans* was grown in minimal media where 100% = no inhibition and 0% = complete inhibition of growth. Potential impact of residual organic solvent is ruled out by the observation that growth in 2.5% v/v DMSO in media is greater than 96% of that of the control. Values stated are the average of triplicate measurements (*n* = *3*)

The inhibitory effects of all silver(I) complexes **4–6** and silver(I) perchlorate at the test range were superior to those of the corresponding free ligands, the enhanced activity was particularly noteworthy at lower concentrations (*P* < 0.0001 between each complex ligand pair at 30 and 15 M dosages). Although all of the silver(I) compounds were too active over the concentration range studied to allow minimal inhibitory concentrations MIC_80/50_ to be determined it is clear that there is no significant difference between the potency of AgClO_4_ and the three silver(I)-phen-oxazine complexes under the conditions of the experiment (*P* > 0.05 between all silver(I) salts at all dosages). MIC_80_ values for the silver(I) salts are < 15 μM, (Tables 1** and S2, **Fig. 6**)**.

A comparison of the activity of the free ligands and their corresponding complexes indicated that complexation to Ag(I) meaningfully enhanced the activity of all ligands against *C. albicans* (*P* ≤ 0.0001 between each complex/ligand pair, at 30 and 15 M). Whilst most evident at 30 M dosages, the relative effectiveness of the free ligands correlated with the lipophilicity of their ester hydrocarbon tails the difference in antifungal behaviour of the corresponding phen-oxazine complexes in minimal media at the concentrations studied was not influenced by ligand lipophilicity (*P* > 0.05 between all complexes at all dosages). It can therefore be concluded that, in the context of their antifungal activity, for this series of phenanthroline derived ligands molecular design involving complexation to silver(I) vastly out-weighed the lipophilic effect. This result mirrors that previously recorded for the perchlorates of Ag(phendione)_2_ and Ag(phen)_2_ which were significantly more active than the corresponding metal free ligands (MIC values for in vitro anti-*Candida* susceptibility 0.5 and 8.8 μM for the complexes, and 3 and 14 μM for the free ligands respectively) (McCann et al. 2004).

The efficacy of antifungal agents is known to be influenced by media composition (Klepser et al. 1997; Muller et al. 2001). In one recent in vitro study, drug activity against central nervous system fungal pathogens, activity in sabouraud-dextrose broth differed markedly compared to cerebeospinal fluid media (al Jalali et al. 2019). In another investigation, it was noted that growth, adhesion and biofilm formation of *Candida* species was profoundly affected by choice of media culture (Weerasekera et al. 2016). Consequently, we were interested in seeing how media composition influenced the inhibitory potential of our test compounds against *C. albicans.* A series of experiments conducted in nutrient rich media (YEPD) afforded dramatically different results to those generated from the study using minimal media. Over the concentration test range (15–60 μM), none of the ligands (**1–3**), silver(I) perchlorate or the silver(I) complexes (**4–6**) showed any growth inhibition when the fungal cells were cultivated in YEPD (**Table S3**, **Row A**). These results clearly showed that media choice can be a key determinant of the outcome of a study of the antifungal activity of a test compound against *C. albicans*. The observed differences in activity of the silver(I) complexes between minimal and nutrient-rich media could relate to differing dynamic behaviour in the two media. Ligand exchange reactions are possible, whereby the central silver(I) ion loses its phen-oxazine ligands and becomes ligated by donor atoms present in the peptone and yeast extract of the YEPD. If the metal ion is encapsulated and trapped by the proteins it may not be available for interaction with the fungal cells.

**Fig. 7:**
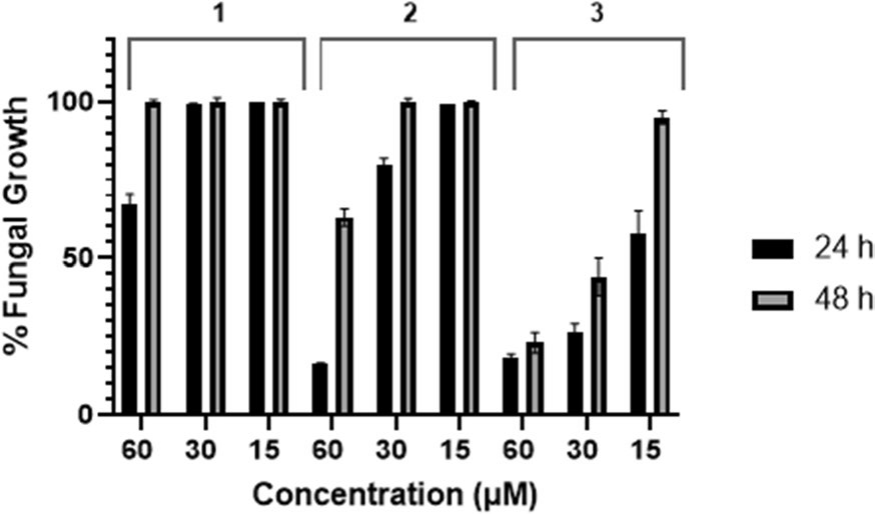
Antifungal activity of ligands **1**–**3** over an extended time, measured as their ability to restrict *C. albicans* growth in minimal media, at 15–60 M dosages, after 24 and 48 h incubation

With an awareness of the dynamic solution behaviour of the complexes **4–6**, and given that incubation time has previously been noted as one parameter influencing the behaviour of known antifungal agents (Nguyen and Yu 1999), it was important to learn more about the initiation and the duration of effectiveness of the test compounds against *C. albicans*. Following incubation in minimal media, plates were read between 3 and 48 h (Tables 2 and** S4**). At the 3 h point for samples treated with ligands **1–3** growth inhibition was less than 10%. Between 3 and 6 h the antimicrobial effect started to become apparent, however, it was only after 24 h that key differences in potency of the test compounds emerged. Whilst each of the free ligands showed dose-dependent, maximum potency after 24 h their activities dipped to varying extents after 48 h (Fig. 7). After 48 h, irrespective of dosage, the methoxy ester **1** was completely ineffective in controlling fungal growth. Activity from the propyl analogue **2** after 48 h was evident only at the 60 μM dose albeit it significantly reduced relative to that observed at 24 h (*P* < 0.0001 between 24 h (17% growth) and 48 h (64% growth), 60 μM dose). Although after 48 h incubation full growth returned to plates dosed at 15 μM with the hexyl ester ligand **3,** at higher dosages it continued to show antifungal activity with growth limited, respectively, to 45% (30 μM) and 24% (60 μM) of the control. Inhibition of *C. albicans* growth in the presence of **3** is statistically different to that of analogues **1** and **2** at 30 and 60 mM dosages (*P* < 0.0001 for all comparisons between **1–3**, at 48 h in this doesage range). Clearly over the 24–48 h window, both dosage and lipophilicity impact on fungal inhibition; ligand **3** with the longer alkyl chain was not only more effective but also a longer lasting antifungal agent than the less lipophilic analogues **1** and **2**.

**Table 2:** In vitro inhibitory effects of test samples determined as their ability, after incubation for time periods up 48 h, to limit the growth of *C. albicans* in in minimal media

Incubation time	Test sample						
	Ligands			Silver(I) salts			
	1	2	3	4	5	6	AgClO_4_
	Percentage Growth^#^ at 60 μM, 30 μM and 15 μM dosage levels						
3 h	90, 93,95%	93, 93, 94%	100, 100, 100%	100, 100, 97%	100, 100, 100%	100, 100, 100%	100, 90, 93%
24 h	68, 99, 100%	17, 80, 100%	18, 27, 57%	15, 12, 11%	12, 10, 9%	11, 12, 14%	9, 10, 12%
48 h	100, 100, 100%	64, 100, 100%	24, 45, 95%	19, 19, 17%	13, 10, 9%	15, 17, 21%	11, 91, 100%

^#^Percentage growth is measured relative to a control sample, where 100% = no inhibition, and 0% = complete inhibition of growth. Potential impact of residual organic solvent is ruled out by the observation that growth in 2.5% v/v DMSO in media is greater than 96% of that of the control. Values stated are the average of triplicate measurements (*n* = *3*)

When the study was extended to include the silver(I) salts important differences emerged in duration and effectiveness of activity between the complexes **4–6**, their corresponding ligands and silver perchlorate. In sharp contrast to the metal-free ligands, for all three silver(I) phen-oxazine complexes, across all dosages (60–15 μM), there was no significant reduction in activity at 48 h, with fungal growth continuing to be limited to about 20% of the control (*P* > 0.05 for each complex between 24 and 48 h). It was apparent that, even after 48 h incubation, the difference in lipophilicity of the ligands chelated to silver(I) had no influence on the inhibitory potential of the complexes at the concentrations studied (*P* > 0.05 data compared between any complex pair). It was also noteworthy that, whilst the inhibitory effect of silver(I) perchlorate was similar to that of the phen-oxazine silver(I) complexes at 24 h (*P* > 0.05 data compared between any pair of silver(I) salts at any dosage, 24 h), as the test period was extended AgClO_4_ ceased to be effective at dosages below 60 μM. This study shows that under the conditions of our study, over an extended time period, the antifungal activity of the silver(I) phen-oxazine complexes **4–6** was maintained independent of the relative lipophilicity of the constituent ligands. Furthermore, despite their equivalent potency at 24 h, after 48 h the activity of the silver(I) phen-oxazine complexes at the lower dosage levels, 30 and 15 μM, was hugely superior to that of simple silver(I) perchlorate (*P* < 0.001 between AgClO_4_ and any of **4–6** at dosages less than or equal to 30 μM, 48 h).

Standard protocols for antimicrobial testing generally involve using freshly prepared solutions of the test compounds. In keeping with our observation of dynamic solution behaviour of the silver(I) complexes **4–6** in DMSO, and the apparent instability of complex **6** in the minimal media, it was important to compare the antifungal activity of standing solutions with freshly prepared solution samples. Stock solutions (120 μM) of complexes **4–6** and silver(I) perchlorate were prepared in minimal media containing 5% v/v DMSO. These solutions were stored in the dark at 25 °C. Over a time period of 0–72 h, aliquots were routinely withdrawn and a series of growth experiments were conducted. In each experiment, fungal cells were treated with a solution of the test compound (which had been stored for the specified time period) and fungal growth measured after a 24 h incubation period (Table 3 and S5-S11). At all doses studied, irrespective of the duration of prior standing in solution, the inhibitory effects of the test compounds from the stored solutions were similar to those of the freshly prepared solutions in minimal media (*P* > 0.05 for all **4–6** and **AgClO**_**4**_ pairings across the 0–72 h pre-test standing period). As was the case with the freshly prepared solutions, no activity was observed for any compound following standing in nutrient-rich YEPD media across the 0–72 h storage period (**Table S3, Rows B-F**). These findings support the validity of biological testing of silver(I) salts using test solutions which have been prepared within a 0–72 h time window and which have been stored in the dark at room temperature.

**Table 3:** In vitro inhibitory effects of previously prepared solutions of silver(I) complexes **4–6** and silver(I) perchlorate determined as their ability after incubation for 24 h to limit the growth of *C. albicans* in minimal media*

Time pre-prepared solutions were stored prior to testing	Test sample			
	4	5	6	AgClO_4_
	Percentage growth ^#^ at 60 μM, 30 μM and 15 μM dosage levels			
0 h	15, 12, 11%	12, 10, 9%	11, 12, 14%	9, 10, 12%
3 h	13, 13, 12%	10, 9, 8%	13, 12, 13%	10, 11, 11%
6 h	11, 11, 12%	9, 8, 8%	11, 10, 9%	10, 10, 13%
24 h	13, 13, 13%	12, 11, 10%	14, 12, 19%	11, 11, 27%
48 h	15, 17, 18%	11, 10, 9%	25, 20, 19%	11, 11, 13%
72 h	12, 12, 14%	10, 8, 7%	12, 10, 13%	11, 13, 13%

*When the same experiments were conducted in nutrient-rich YEPD media no growth inhibition was observed for any test compound across the dosage range studied, 60–15 μM (Table S3 rows B-F)

^#^Percentage growth is measured relative to a control sample where 100% growth = no inhibition and 0% = complete inhibition of growth. Potential impact of residual organic solvent is ruled out by the observation that growth in 2.5% v/v DMSO in media is greater than 96% of that of the control. Values stated are the average of triplicate measurements (*n* = *3*)

## Conclusion

In this work three Ag(I) *bis*(phen-oxazine) complexes, with stoichiometry of 1:2 Ag(I):ligand, were synthesised and characterised. All three complexes showed fluxional behaviour with competitive exchange (fast on the NMR time scale) in DMSO-d6 with bidentate but not with monodentate ligands. Evidence for solution instability in biological media was less compelling, and UV–visible spectra over a period up to 72 h in 5% v/v DMSO in minimal media tentatively support aggregation of the most lipophilic complex, **6**. There was insufficient evidence to attribute the biological activity of the complexes to their dynamic behaviour, however, unambiguous differences between the inhibitory effects of the metal-free ligands and the silver(I) complexes against *C. albicans* emerged. In an analysis of biological activity, the importance of the choice of media, the duration of the incubation period and sample preparation was established*.* All of the test compounds were active in minimal media but not in nutrient-rich YEPD media. Within the ligand series, activity correlated with the length of the alkyl chain. However, the enhancement in biological activity that was observed in going from the least **1** to the most lipophilic ligand **3** was outstripped by the enhancement that followed complexation of each ligand to Ag(I). This result makes a compelling case for complexation to silver(I) over increasing ligand lipophilicity as a guiding principle in designing antifungal agents.

No lipophilic effect was observed for the Ag(I) complexes and all three had similar activities to each other and to AgClO_4_ after 24 h incubation. However, after 48 h incubation the simple silver(I) salt became totally inactive at low dosage whilst the silver(I) phen-oxazine complexes held activity at all doses. These results make a case for extending antimicrobial studies beyond the traditional 24 h incubation period. Insignificant differences between the biological activity of freshly prepared and pre-prepared test solutions (which were stored in darkness at room temperature) makes the case for the appropriateness of using test solutions of silver(I) complexes prepared within a 72 h time window.

### Supplementary Information

Below is the link to the electronic supplementary material.

Supplementary file1 (DOCX 3345 KB)
