# Supplementary material for: Synthesis, characterisation, and solution behaviour of Ag(I) bis(phenanthroline-oxazine) complexes and the evaluation of their biological activity against the pathogenic yeast Candida albicans
Source: Biometals. 2023 Jun 28;36(6):1241–56. doi: 10.1007/s10534-023-00513-w (PMC10684714; doi:10.1007/s10534-023-00513-w)
Supplement: Supplementary file 1 — Supplementary file1 (DOCX 3345 KB) [file 10534_2023_513_MOESM1_ESM.docx]

Supporting Information

Synthesis, Characterisation, and Solution Behaviour of Ag(I) *bis*(phenanthroline-oxazine) complexes and the evaluation of their biological activity against the pathogenic yeast *Candida albicans*

Clara Evans^a,b^**,** Muhib Ahmed^a,b^, Darren F. Beirne^a,b^, Malachy McCann^a^, Kevin Kavanagh^d,b^, Michael Devereux^c^, Denise Rooney^a,b*^, Frances Heaney^a,b*^

*^a^* *Department of Chemistry, Maynooth University, Maynooth, Co. Kildare, Ireland.*

*^b^ Kathleen Lonsdale Institute for Human Health Research, Maynooth University, Maynooth, Co. Kildare, Ireland.*

*^c^ The Centre for Biomimetic & Therapeutic Research, Focas Research Institute, Technological University Dublin, City Campus, Camden Row, Dublin 8, Ireland.*

*^d^ Department of Biology, Maynooth University, Maynooth, Co. Kildare, Ireland.*

**Contents**

**Section 1 - Experimental**

**Synthesis of [Ag(L)_2_](ClO_4_)….**………………………………………………….…………………………………………...**4**

**Section 2 – Characterisation data for Ag(I) complexes**

**Figure S1:** FTIR spectrum of **4**………………………………………………….…………………………………………...**5**

**Figure S2:** ^1^H NMR spectrum of **4** in DMSO-d6……………………………………...……………….……….……**5**

**Figure S3:** ^13^C NMR spectrum of **4** in DMSO-d6…………………..………………….………………..…….…….**6**

**Figure S4:** FTIR spectrum of **5**…………..……………………………………………………………………..……………**7**

**Figure S5:** ^1^H NMR spectrum of **5** in DMSO-d6…………………………………………………………...…..……**7**

**Figure S6:** ^13^C NMR spectrum of **5** in DMSO-d6………………………..………………………………………..…**8**

**Figure S7:** FTIR spectrum of **6**……………………………………………………………………………..………………**..9**

**Figure S8:** ^1^H NMR spectrum of **6** in DMSO-d6…………………………………………………………………...**..9**

**Figure S9:** ^13^C NMR spectrum of **6** in DMSO-d6…………………………………………………………..………**10**

**Figure S10:** Isotopic pattern found from high resolution Mass spectrometry compared to simulation for **5**………………………………………………………………………………………………………………….**11**

**Figure S11:** Isotopic pattern found from high resolution Mass spectrometry compared to simulation for **6**………………………………………………………………………………………………………………….**11**

**Section 3 - Job's method of continuous variation to determine the stoichiometry of Ag(I) complexes**

**Figure S12:** ^1^H NMR spectra of relative ratios of AgClO_4_:**1** with a fixed total concentration

of 40 mM in DMSO-d6 at 25 °C……………………………………………….…………………………………………**12**

**Figure S13:** Job’s Plot created for AgClO_4_:**1** solutions from data given in **Figure S12**…………..**12**

**Figure S14**: ^1^H NMR spectra of relative ratios of AgClO_4_:**2** with a fixed total concentration

of 40 mM in DMSO-d6 at 25 °C…………………………………………………………………………………………..**13**

**Figure S15:** Job’s Plot created for AgClO_4_:**2** solutions from data given in **Figure S14** ………….**13**

**Figure S16**: ^1^H NMR spectra of relative ratios of AgClO_4_:**3** with a fixed total concentration

of 40 mM in DMSO-d6 at 25 °C………………………….……………………………………………………………….**14**

**Figure S17:** Job’s Plot created for AgClO_4_:**3** solutions from data given in **Figure S16** ………….**14**

**Section 4 – UV-visible Spectra for Compounds 1-6**

**Figure S18**: UV-visible spectra of **1** in DMSO……….………………………………………………………….….**15**

**Figure S19**: UV-visible spectra of **2** in DMSO…………………………………………………………………….…**15**

**Figure S20**: UV-visible spectra of **3** in DMSO……………………………………………………………………….**16**

**Figure S21**: UV-visible spectra of **4** in DMSO……………………………………………………………………….**16**

**Figure S22**: UV-visible spectra of **5** in DMSO……………………………………………………………………….**17**

**Figure S23**: UV-visible spectra of **6** in DMSO……………………………………………………………………….**17**

**Table S1:** Wavelengths of absorption maxima (λ_max_) and extinction coefficient (ε)

values for **1-6** in DMSO……………………………………………………………………………………………………….**18**

**Figure S24**: UV-visible spectra of **4** in 5% v/v DMSO minimal media monitored 0-72 h……….**18**

**Figure S25**: UV-visible spectra of **5** in 5% v/v DMSO minimal media monitored 0-72 h.………**19**

**Figure S26**: UV-visible spectra of **6** in 5% v/v DMSO minimal media monitored 0-72 h.………**19**

**Section 5 - NMR Data on the Dynamic behaviour of Ag(I) complexes**

**Figure S27**: ^1^H NMR spectra of (a) complex **4** (b) ligand **1** and complex **4** in a 1:1 ratio (c) ligand **1** in DMSO-d6 at 25 °C……………………………………………………………………………………………..**20**

**Figure S28**: ^1^H NMR spectra of (a) complex **4** (b) phenanthroline and complex **4** in a 1:1 ratio (c) phenanthroline in DMSO-d6 at 25 °C…………………………………………..………………………..**21**

**Figure S29**: ^1^H NMR spectra of (a) complex **4** (b) pyridine and complex **4** in a 1:1 ratio (c) pyridine in DMSO-d6 at 25 °C………………………………………………………………………..……………..…...**21**

**Section 6 - Results of Biological Testing**

**Table S2**: *In vitro* testing of inhibitory effects of freshly prepared solutions of compounds **1-6** and AgClO_4_ in minimal media following 24 h incubation………………………………………….…..…….**22**

**Table S3**: *In vitro* testing of inhibitory effects of compounds **1-6** and AgClO_4_ in YEPD media following standing of test compounds in solution in the dark for time periods between 0-72 h ………………………………………………………………………………………………………………………………….…….**22**

**Table S4**: *In vitro* testing of initiation and duration of activity of compounds **1-6** and AgClO_4_ following incubation for time periods from 3-48 h.…………………………………………………….………**23**

**Tables S5-S11**: *In vitro* testing of inhibitory effects of test compounds following standing in minimal media for time periods between 0-72 h ………………………………………………………….**23-25**

- **Table S5:** Inhibitory effect of ligand **1** …………………………………………….……………….….…**23**
- **Table S6**: Inhibitory effect of ligand **2**..…………………………………………..………………………**24**
- **Table S7**: Inhibitory effect of ligand **3** ……………………………………………………………..…….**24**
- **Table S8**: Inhibitory effect of AgClO_4_ ……………………………………………………….... ...........**24**
- **Table S9**: Inhibitory effect of Complex **4** …………………………………………………………….….**25**
- **Table S10**: Inhibitory effect of Complex **5** ………………………………………………………………**25**
- **Table S11**: Inhibitory effect of Complex **6** ……………………………………………………………...**25**

**Synthesis of** **[Ag(L)_2_](ClO_4_) 5 and 6**

A 10 mL ACN solution of AgClO_4_ (0.054 g, 0.26 mmol) was added to 90 mL of heated ACN containing **L** (0.52 mmol). The solution was heated at reflux for 2 hours in the absence of light. After cooling to room temperature, the resulting yellow solution was reduced *in vacuo* to ~ 5 mL and the product was precipitated in 400 mL of diethyl ether. The yellow precipitate was filtered and washed with 3 x 50 mL portions of diethyl ether and dried under vacuum.

**Complex 5, L** = **2**, Yield = 0.191 g, 75%.

**Complex 6, L** = **3**, Yield = 0.148 g, 63%.

**Section 2 - Characterisation data for Ag(I) complexes**


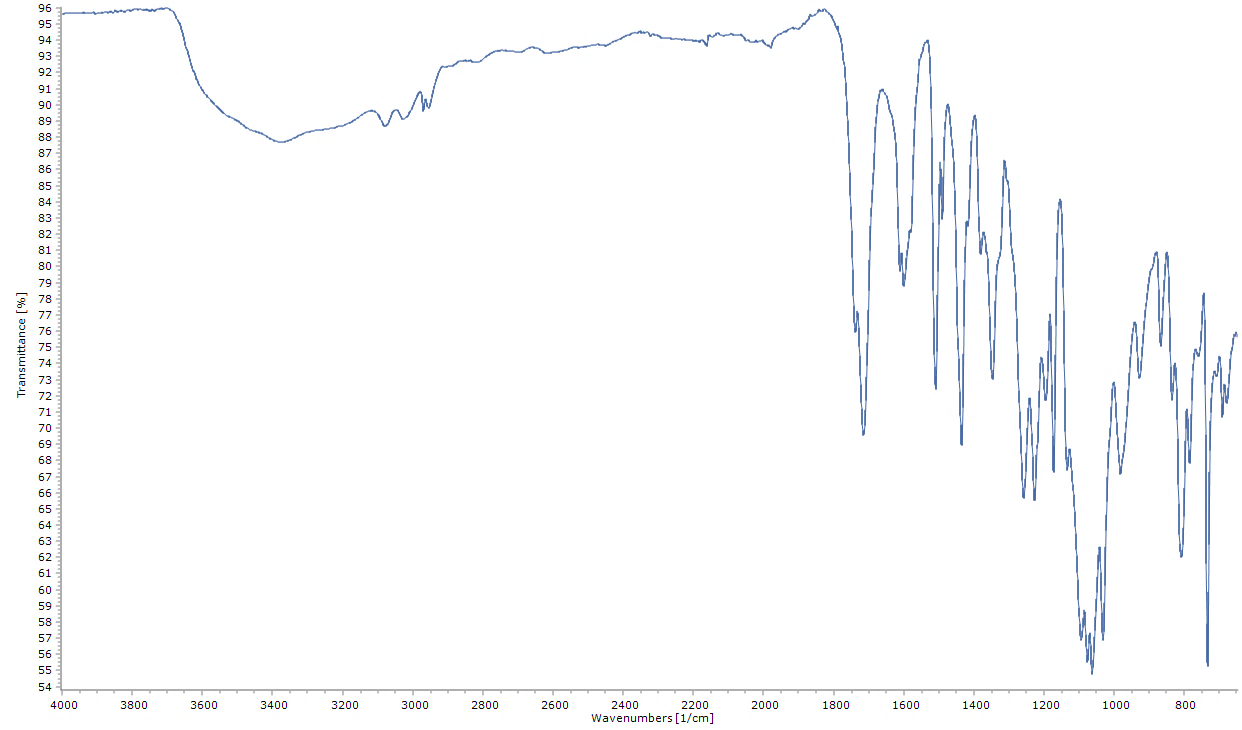


**Figure S1:** FTIR (ATR, cm^-1^) spectrum of **4**: 3375, 1715, 1611, 1600, 1509, 1435, 1347, 1258, 1173, 1095, 1031, 809, 733.


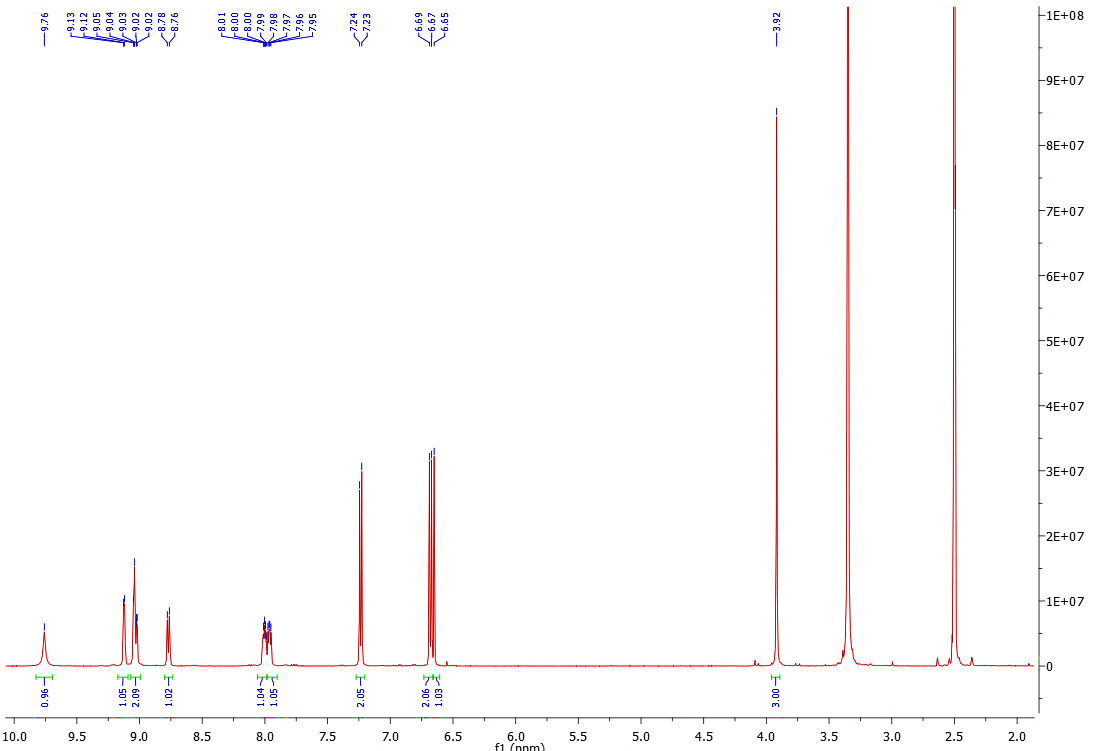


**Figure S2:** ^1^H NMR spectrum of **4** in DMSO-d6.

**^1^H NMR** (DMSO-d6, 500 MHz): **δ** 9.76 (br s, 1H, O**H**), 9.12 (d, *J* = 4.4 Hz, 1H, phen**H**),

9.05 – 9.02 (m, 2H, phen**H**), 8.77 (d, *J* = 8.2 Hz, 1H, phen**H**), 8.01 – 7.99 (m, 1H,

phen**H**), 7.96 (dd, *J* = 4.5, 7.7 Hz, 1H, Phen**H**), 7.23 (d, *J* = 8.7 Hz, 2H, Ar**H**), 6.68 (d, *J* = 8.7 Hz, 2H, Ar**H**), 6.65 (s, 1H, oxazine-C**H**), 3.92 (s, 3H, C**H_3_**).

**
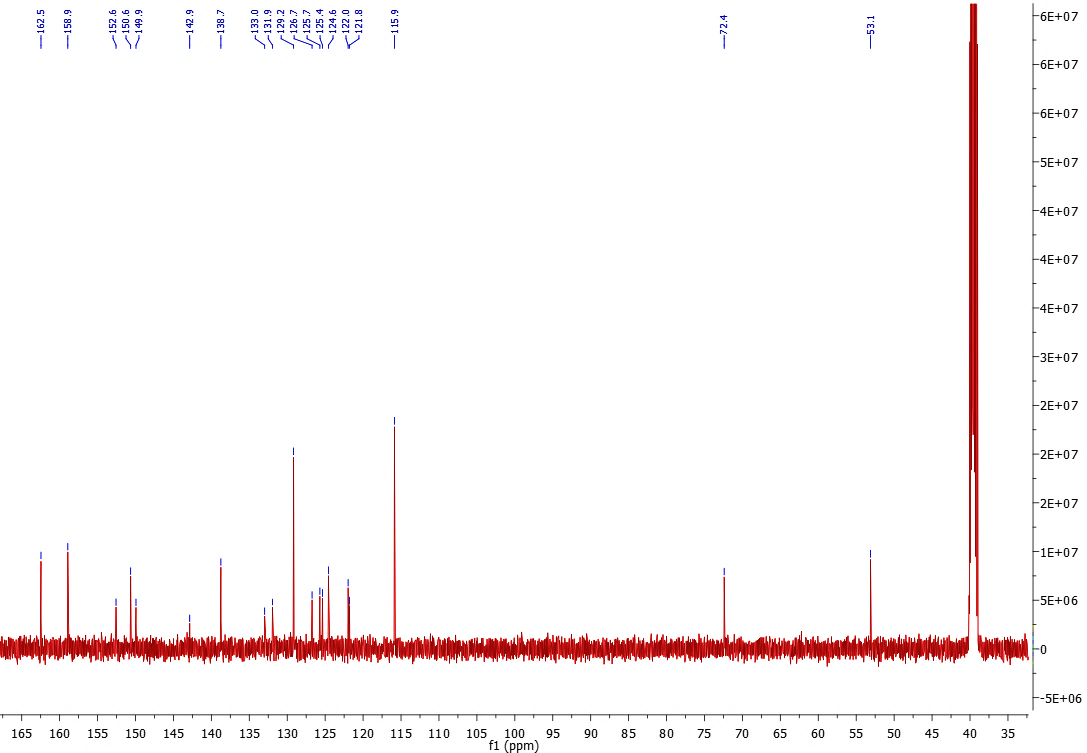
**

**Figure S3:** ^13^C NMR spectrum of **4** in DMSO-d6.

**^13^C NMR** (DMSO-d6, 126 MHz): **δ** 162.5 (**C**=O), 158.9 (**C**-OH), 152.6 (phen**C**-H), 150.6 (oxazine-**C**=N), 149.9 (phen**C**-H), 142.9 (phen**C**), 138.7 (oxazine-**C**O), 133.0 (phen**C**-H), 131.9 (phen**C**-H), 129.2 (Ar**C**-H), 126.7 (oxazine-**C**N), 125.7 (phen**C**-H), 125.4 (phen**C**-H), 124.6 (phenC), 122.0 (phen**C**), 121.8 (phen**C**), 115.9 (Ar**C-H**), 72.4 (oxazine-**C**H), 53.1 (**C**H_3_).


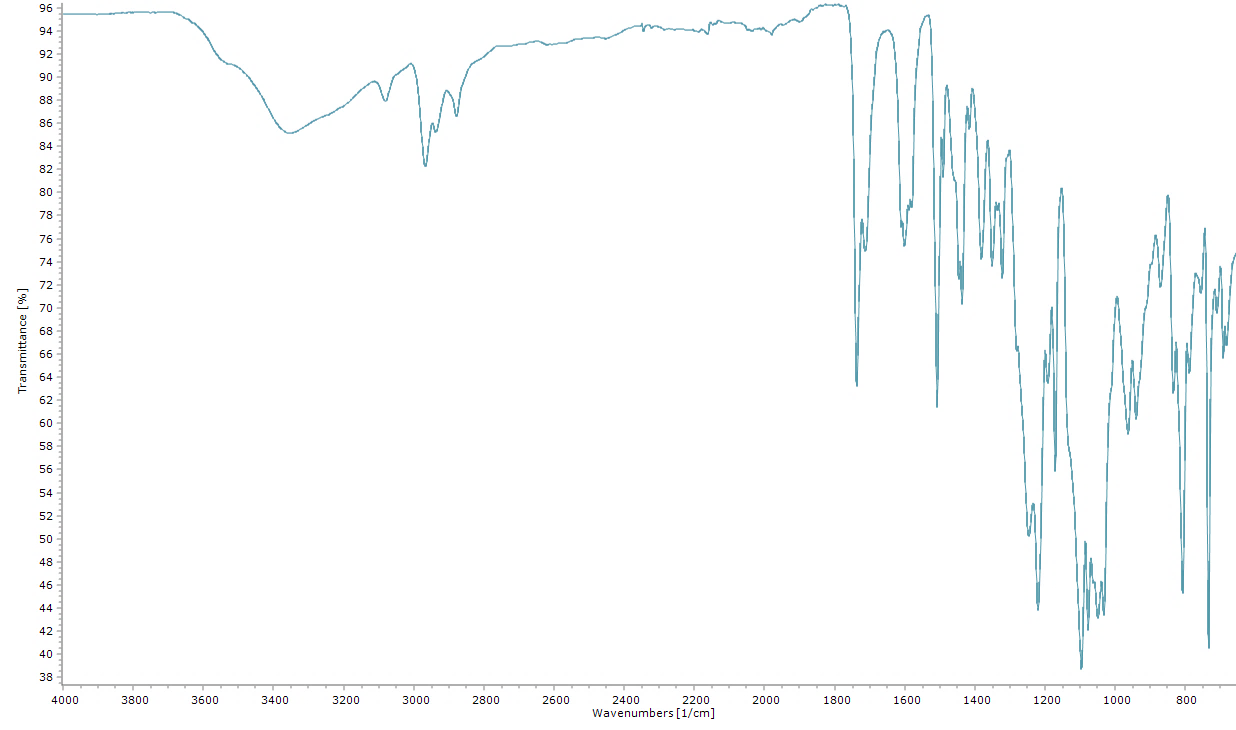


**Figure S4:** FTIR (ATR, cm^-1^) of **5**: 3356, 1737, 1601, 1508, 1437, 1220, 1097, 963, 808, 734.


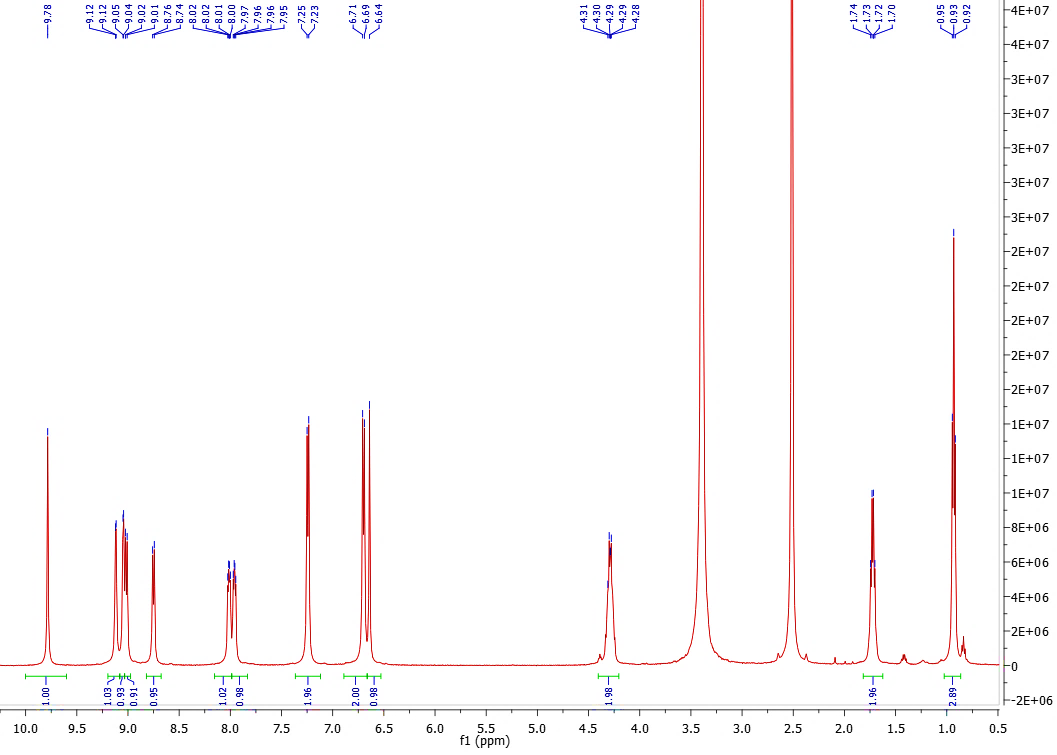


**Figure S5:** ^1^H NMR spectrum of **5** in DMSO-d6.

**^1^H NMR** (DMSO-d6, 500 MHz): **δ** 9.78 (br s, 1H, O**H**), 9.12 (d, *J* = 3.1 Hz, 1H, phen**H**), 9.05 (d, *J* = 3.2 Hz, 1H phen**H**), 9.02 (d, *J* = 8.2 Hz, 1H, phen**H**), 8.75 (d, *J* = 7.9 Hz, 1H, phen**H**), 8.01 (dd, *J* = 4.4, 7.9 Hz, 1H, phen**H**), 7.96 (dd, *J* = 4.4, 8.0 Hz, 1H, phen**H**), 7.24 (d, *J* = 8.4 Hz, 2H, Ar**H**), 6.70 (d, *J* = 8.5 Hz, 2H, Ar**H**), 6.64 (s, 1H, oxazine‑C**H**), 4.33 – 4.24 (m, 2H, -OC**H_2_**), 1.74 – 1.70 (m, 2H, C**H_2_**), 0.93 (t, *J* = 7.3 Hz, 3H, C**H_3_**).


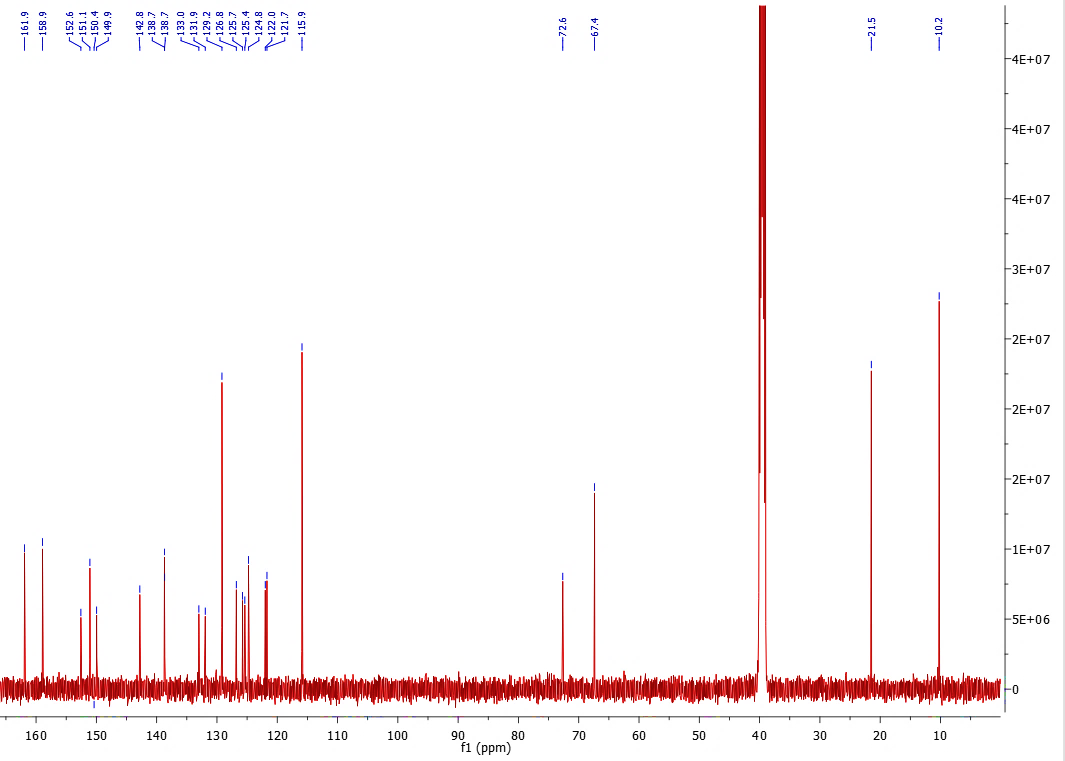


**Figure S6:** ^13^C NMR spectrum of **5** in DMSO-d6.

**^13^C NMR** (DMSO-d6, 126 MHz): **δ** 161.9 (**C**=O), 158.9 (**C**-OH), 152.6 (phen**C**-H), 151.1 (oxazine-**C**=N), 149.9 (phen**C**-H), 142.8 (phen**C**), 138.7 (oxazine-**C**O), 138.7 (Ar**C**), 133.0 (phen**C**-H), 131.9 (phen**C**-H), 129.2 (Ar**C**-H), 126.8 (oxazine-**C**N), 125.7 (phen**C**-H), 125.4 (phen**C**-H), 124.8 (phen**C**), 122.0 (phen**C**), 121.7 (phen**C**), 115.9 (Ar**C**-H), 72.6 (oxazine**C-**H), 67.4 (O**C**H_2_), 21.5 (**C**H_2_), 10.2 (**C**H_3_).


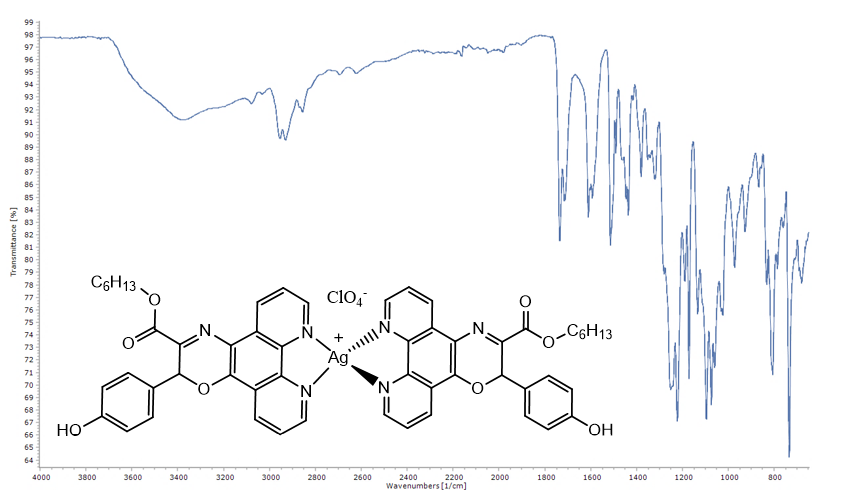


**Figure S7:** FTIR (ATR, cm^-1^) of **6**: 3386, 1735, 1611, 1514, 1436, 1380, 1222, 1096, 807, 734.


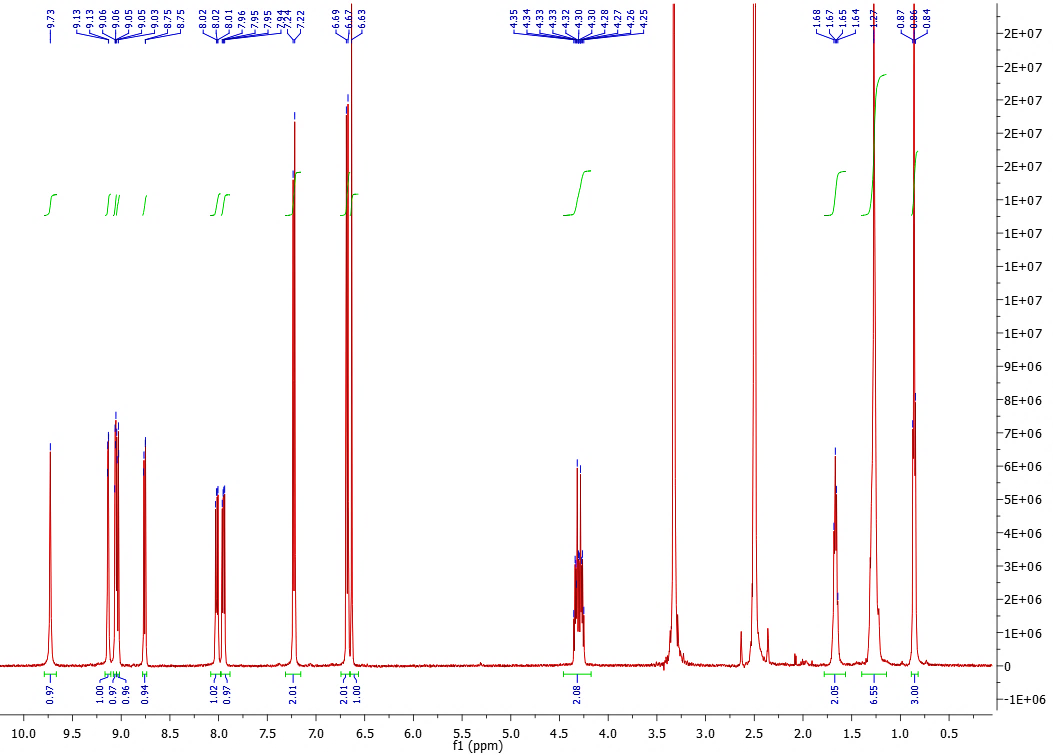


**Figure S8:** ^1^H NMR spectrum of **6** in DMSO-d6.

**^1^H NMR** (DMSO-d6, 500 MHz): δ 9.73 (s, 1H, O**H**), 9.13 (dd, *J* = 1.6, 4.5 Hz, 1H, phen**H**), 9.05 (dd, *J* = 1.5, 4.4 Hz, 1H, phen**H**), 9.0 (dd, *J* = 1.6, 8.3 Hz 1H, phen**H**), 8.76 (dd, *J* = 1.6, 8.3 Hz, 1H, phen**H**), 8.02 (dd, *J* = 4.5, 8.3 Hz, 1H, phen**H**), 7.95 (dd, *J* = 4.5, 8.3 Hz, 1H, phen**H**), 7.23 (d, *J* = 8.7 Hz 2H, Ar**H**), 6.68 (d, *J* = 8.7 Hz, 2H, Ar**H**), 6.63 (s, 1H, oxazine-C**H**), 4.35 – 4.25 (m, 2H, -OC**H**_2_), 1.68 – 1.64 (m, 2H, C**H**_2_), 1.29 – 1.27 (m, 6H, 3 x C**H**_2_), 0.86 (t, *J* = 6.8 Hz 3H, C**H**_3_).

**
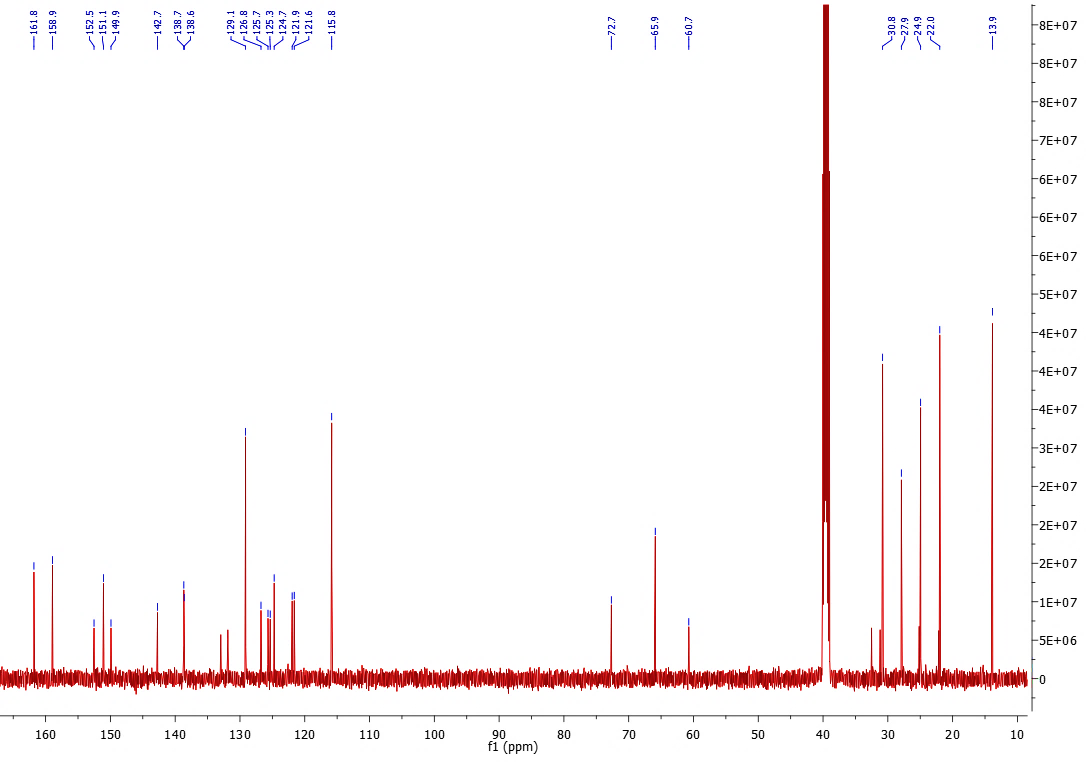
**

**Figure S9:** ^13^C NMR spectrum of **6** in DMSO-d6.

**^13^C NMR** (DMSO-d6, 126 MHz): **δ** 161.8 (**C**=O), 158.9 (**C**-OH), 152.5 (phen**C**-H), 151.1 (oxazine-**C**=N), 149.9 (phen**C**-H), 142.7 (phen**C**), 138.7 (oxazine-**C**O), 138.3 (Ar**C**), 133.0 (phen**C**-H), 131.9 (phen**C**-H), 129.1 (Ar**C**-H), 126.8 (oxazine-**C**N), 125.7 (phen**C**-H), 125.3 (phen**C**-H), 124.7 (phen**C**), 121.9 (phen**C**), 121.6 (phen**C**), 115.8 (Ar**C**-H), 72.7 (oxazine-**C**H**),** 65.9 (O**C**H_2_), 30.8 (OCH_2_**C**H_2_), 27.9 (**C**H_2_), 24.9 (**C**H_2_), 22.0 (**C**H_2_), 13.9 (**C**H_3_).


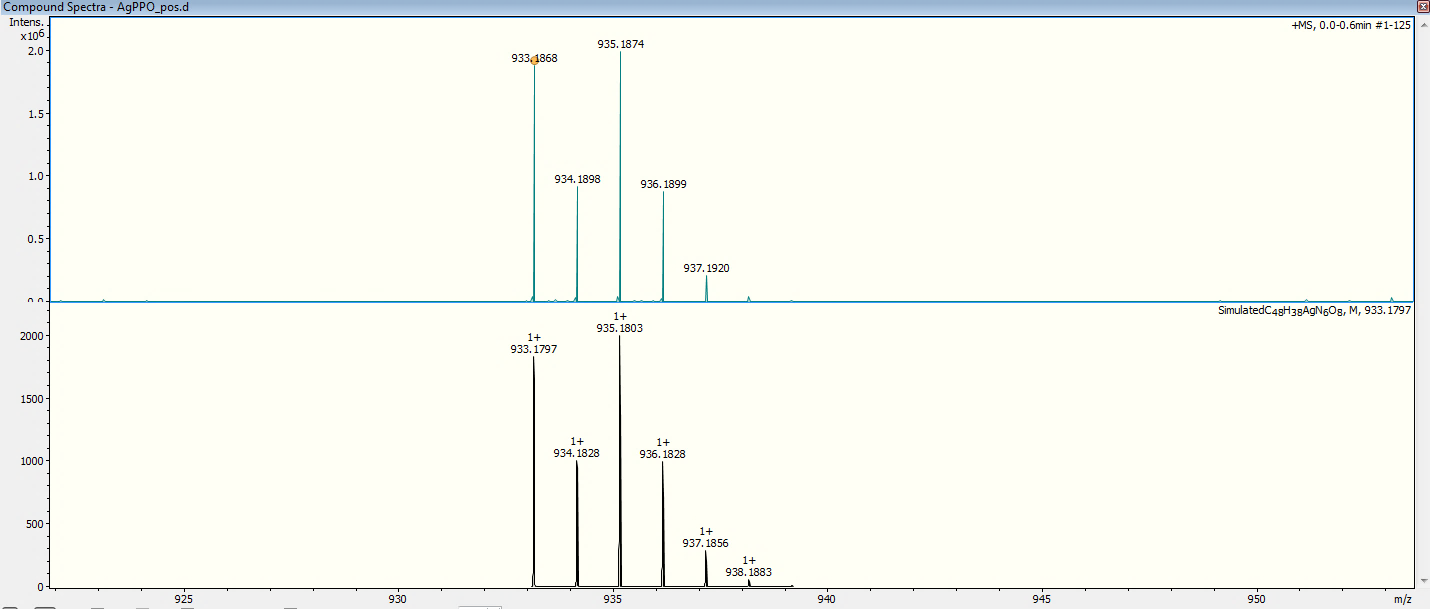


**Figure S10:** ESI Mass spectrum recorded in positive ionisation mode showing the isotopic pattern (top) and simulation of isotopic pattern for **5** (bottom).


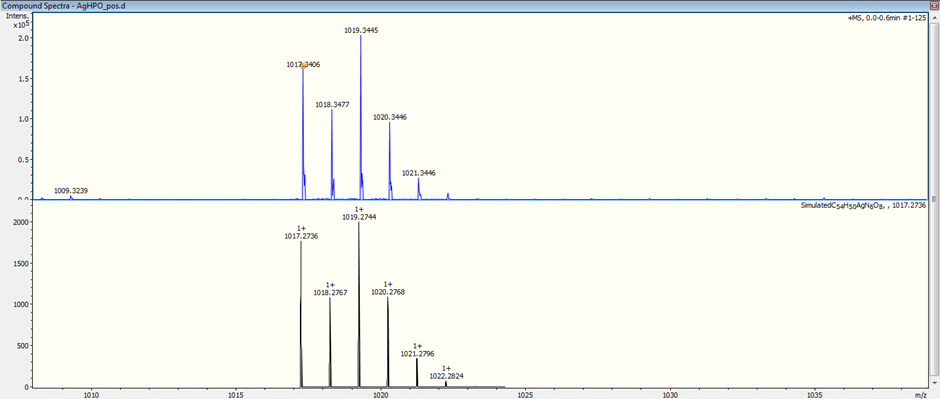


**Figure S11:** ESI Mass spectrum recorded in positive ionisation mode showing the isotopic pattern (top) and simulation of isotopic pattern for **6** (bottom).

**Section 3 - Job's method of continuous variation to determine the stoichiometry of Ag(I) complexes**


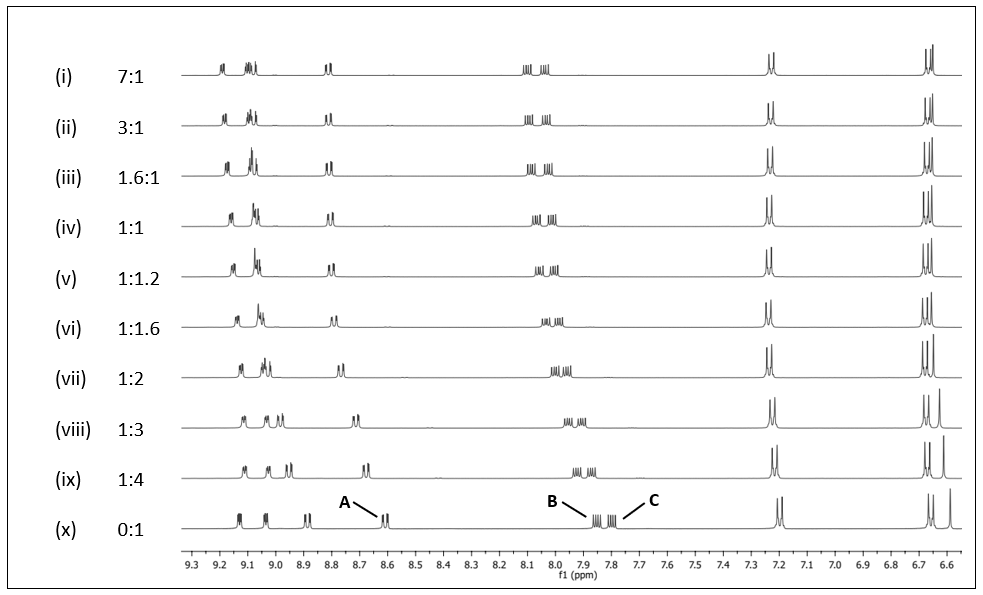


**Figure S12**: ^1^H NMR spectra of relative ratios of AgClO_4_:**1** with a fixed total concentration of 40 mM in DMSO-d6 at 25 °C, varying from (i) 7:1 (AgClO_4_:**1**) to (x) 0:1 (AgClO_4_:**1**).

**Figure S13:** Job’s Plot created from data given in **Figure S12** of various AgClO_4_:**1** solutions with a fixed total concentration of 40 mM in DMSO-d6 at 25 °C, where X_L_ is the mole fraction of methyl ligand **1**, Δδ*X_L_ is the difference in chemical shift (with reference to free ligand) multiplied by the mole fraction of the ligand.

**
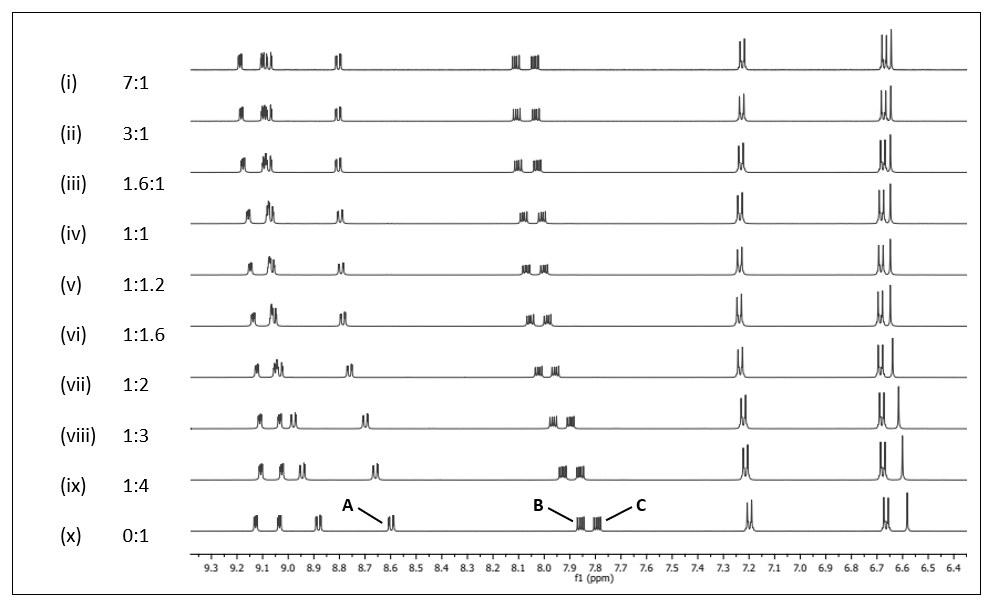
**

**Figure S14:** ^1^H NMR spectra of relative ratios of AgClO_4_:**2** with a fixed total concentration of 40 mM in DMSO-d6 at 25 °C, varying from (i) 7:1 (AgClO_4_:**2**) to (x) 0:1 (AgClO_4_:**2**).

**Figure S15:** Job’s Plot created from data given in **Figure S14** of various AgClO_4_:**2** solutions with a fixed total concentration of 40 mM in d6-DMSO at 25 °C, where X_L_ is the mole fraction of propyl ligand **2**, Δδ*X_L_ is the difference in chemical shift (with reference to free ligand) multiplied by the mole fraction of the ligand.


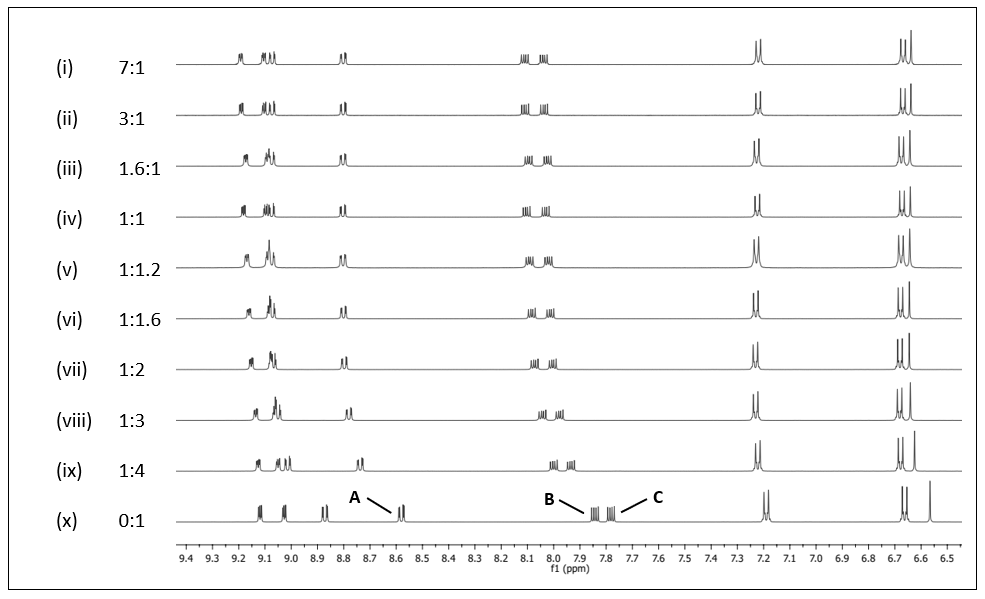


**Figure S16**: ^1^H NMR spectra of relative ratios of AgClO_4_:**3** with a fixed total concentration of 40 mM in DMSO-d6 at 25 °C, varying from (i) 7:1 (AgClO_4_:**3**) to (x) 0:1 (AgClO_4_:**3**).

**Figure S17:** Job’s plot of various AgClO_4_:**3** solutions with a fixed total concentration of 40 mM in DMSO-d6 at 25 °C, where X_L_ is the mole fraction of hexyl ligand **3**, Δδ*X_L_ is the difference in chemical shift (with reference to free ligand) multiplied by the mole fraction of the ligand.

**Section 4 - UV-visible Spectra for Compounds 1-6**


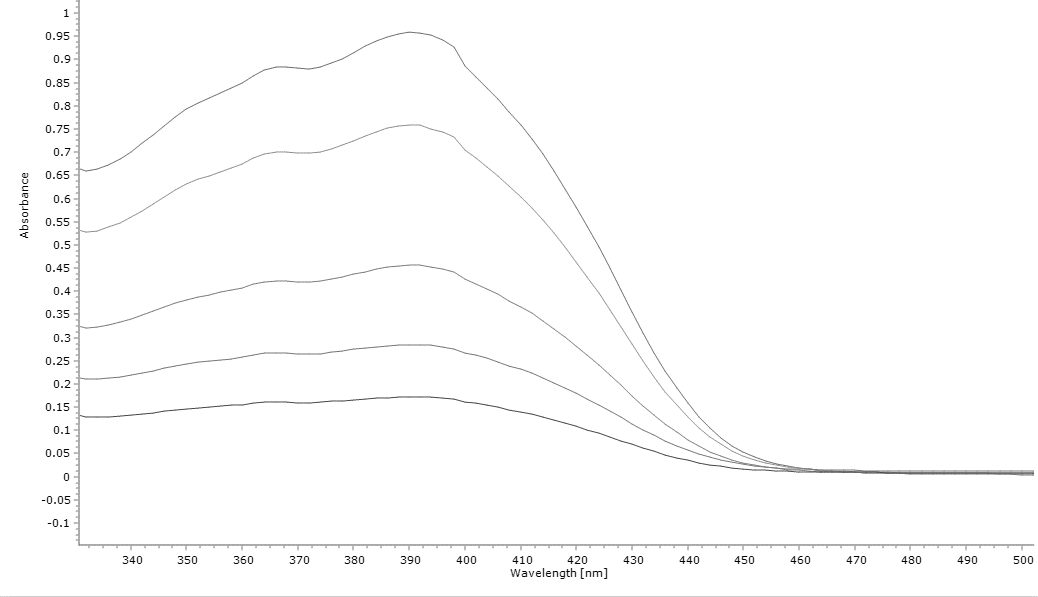


**Figure S18**: UV-visible spectra of **1** at concentrations of 2.16 x 10^-5^ – 1.25 x 10^-4^ M in DMSO used to determine the extinction coefficient (ε). ε = 7,646.6 M^-1^ cm^-1^.


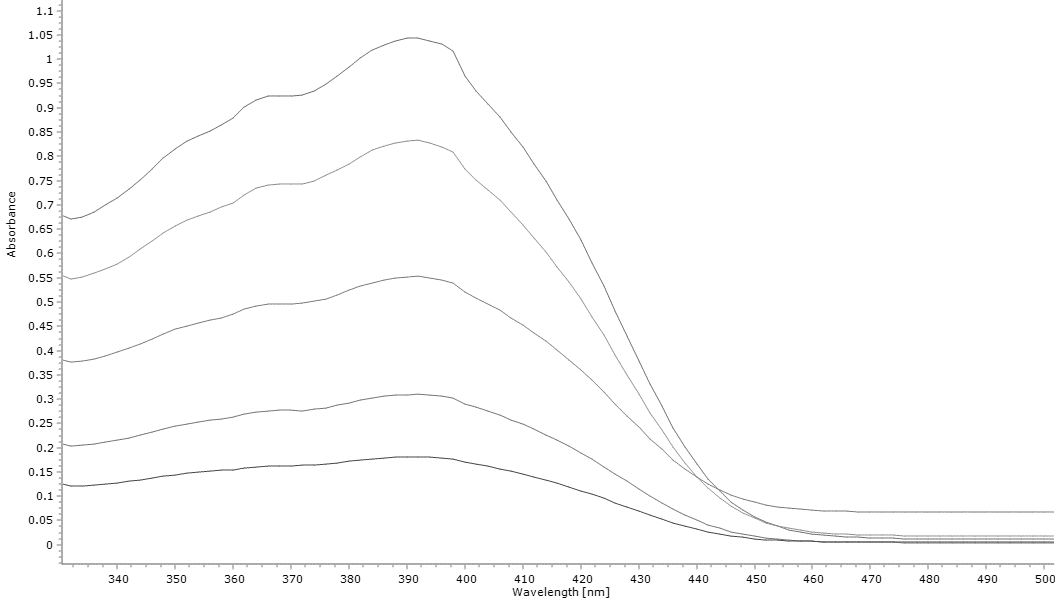


**Figure S19**: UV-visible spectra of **2** at concentrations of 2.36 x 10^-5^ – 1.37 x 10^-4^ M in DMSO used to determine the extinction coefficient (ε). ε = 7,717 M^-1^ cm^-1^.


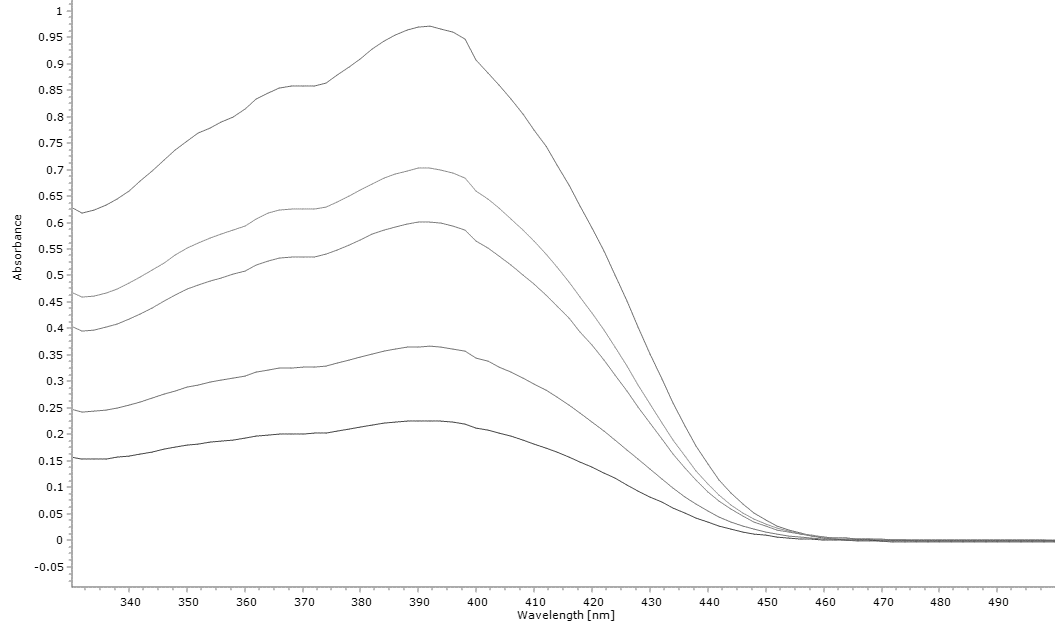


**Figure S20**: UV-visible spectra of **3** at concentrations of 2.75 x 10^-5^ – 1.33 x 10^-4^ M in DMSO used to determine the extinction coefficient (ε). ε = 7,464.7 M^-1^ cm^-1^.


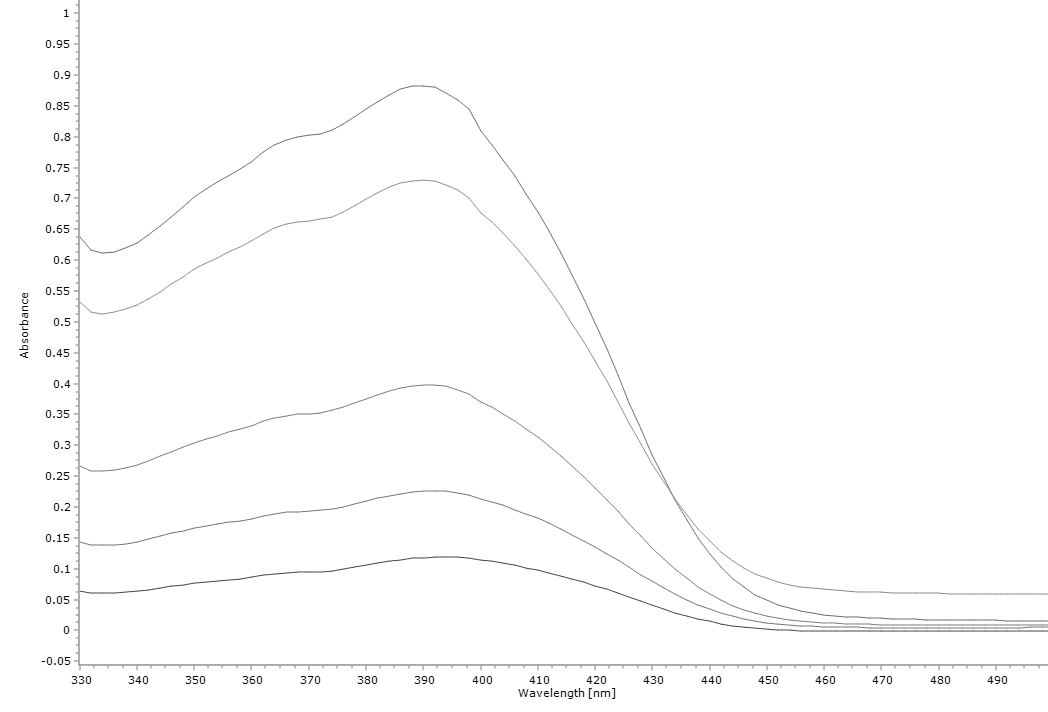


**Figure S21**: UV-visible spectra of **4** at concentrations of 1.15 x 10^-5^ – 6.67 x 10^-5^ M in DMSO used to determine the extinction coefficient (ε). ε = 13,172 M^-1^ cm^-1^.

**
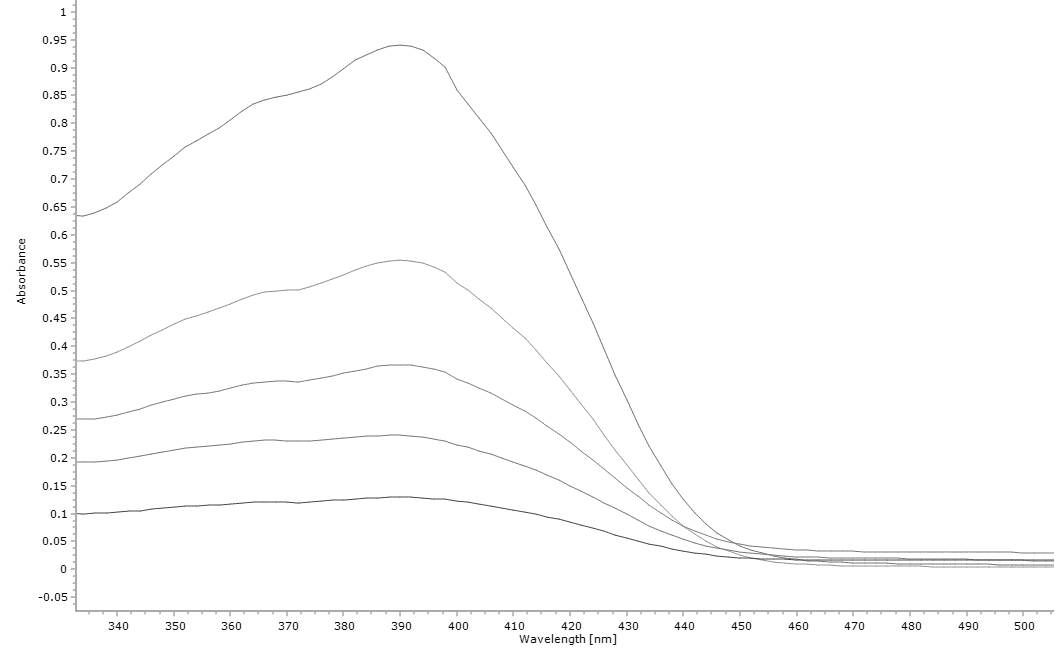
**

**Figure S22**: UV-visible spectra of **5** at concentrations of 7.85 x 10^-6^ – 6.06 x 10^-5^ M in DMSO used to determine the extinction coefficient (ε). ε = 15,629 M^-1^ cm^-1^.


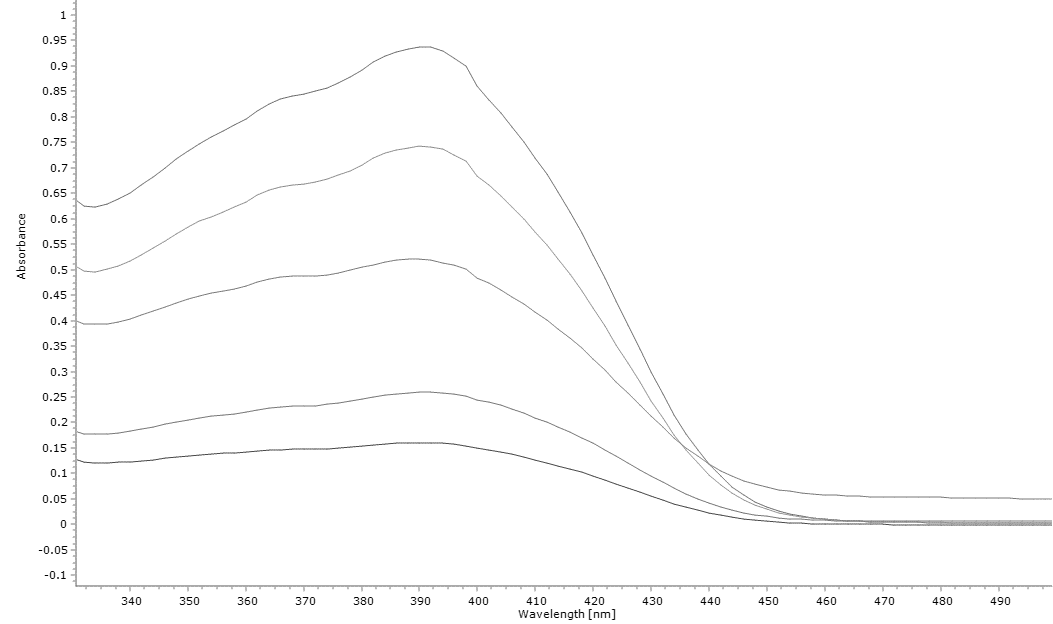


**Figure S23**: UV-visible spectra of **6** at concentrations of 1.03 x 10^-5^ – 6.00 x 10^-5^ M in DMSO used to determine the extinction coefficient (ε). ε = 15,877 M^-1^ cm^-1^.

**Table S1:** Wavelengths of absorption maxima (λ_max_) and extinction coefficient (ε) values for ligands **1**-**3** and silver(I) complexes (**4**-**6**) in DMSO.

| Ligand | Silver(I) complex |
| --- | --- |
| λ_max_ (ε)  nm (M^-1^ cm^-1^) | λ_max_ (ε)  nm (M^-1^ cm^-1^) |
| **1**  389 (7,646)  **2** 390 (7,717)  **3**  390 (7,464) | **4**  389 (13,172)  **5** 390 15,629)  **6** 391 (15,877) |


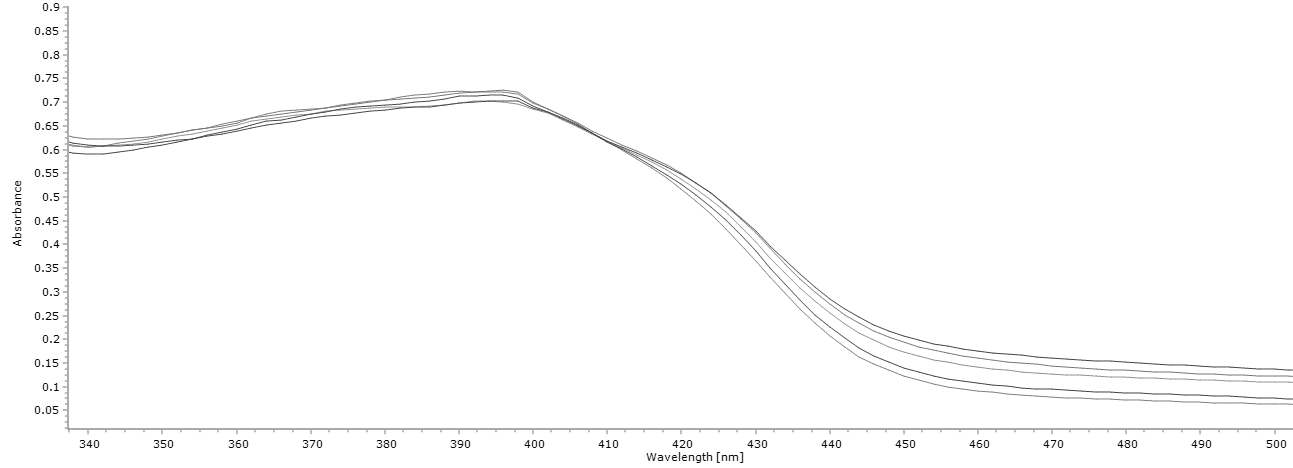


**Figure S24:** UV-visible spectra of a 60 µM solution of **4** in 5% v/v DMSO in minimal media monitored from 0-72 hours on standing at ambient temperature in the dark.


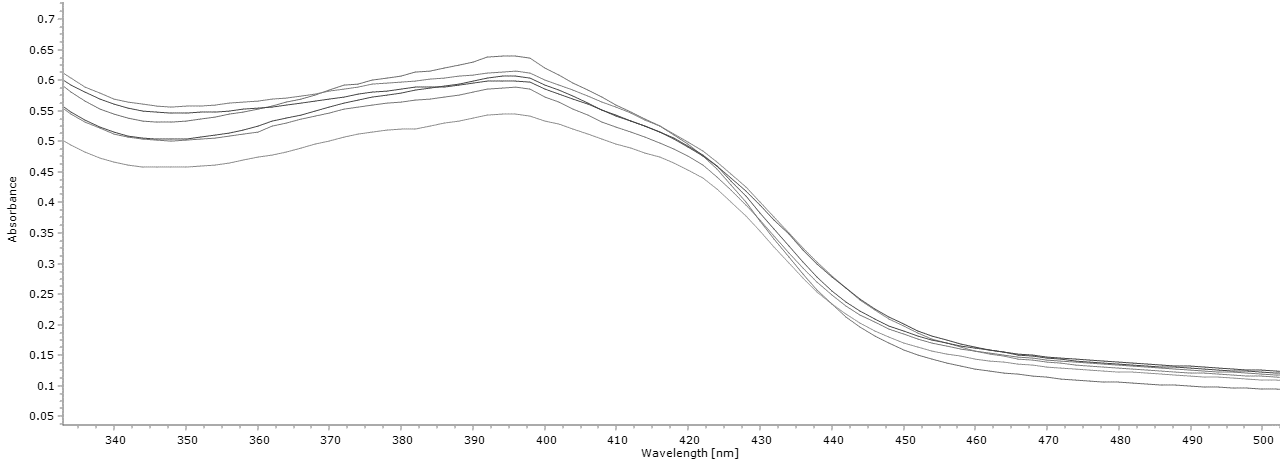


**Figure S25:** UV-visible spectra of a 60 µM solution of **5** in 5% v/v DMSO in minimal media monitored from 0-72 hours on standing at ambient temperature in the dark.


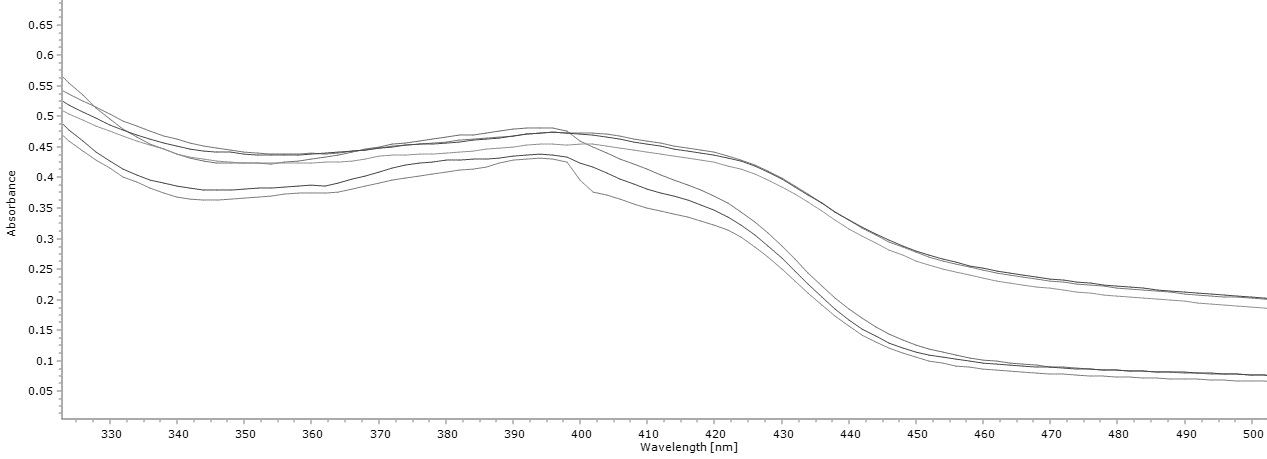


**Figure S26:** UV-visible spectra of a 60 µM solution of **6** in 5% v/v DMSO in minimal media monitored from 0-72 hours on standing at ambient temperature in the dark.

**Section 5 - NMR Data on the Dynamic behaviour of Ag(I) complexes**


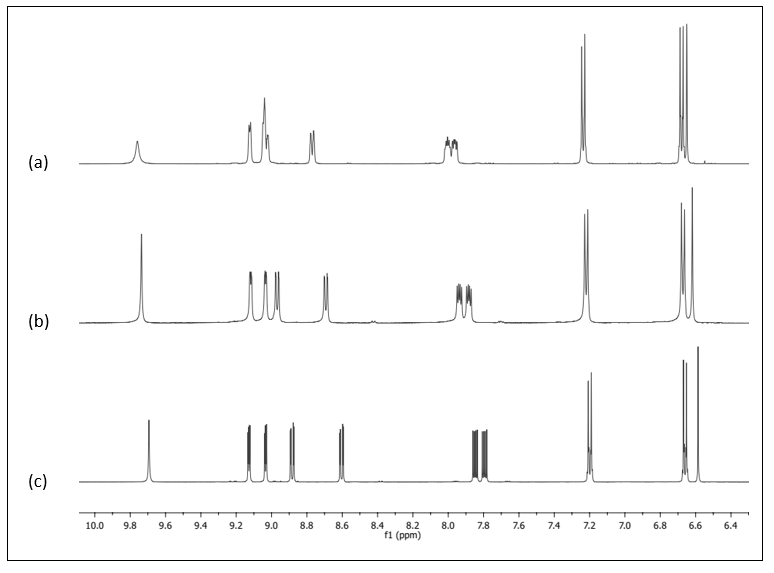


**Figure S27**: ^1^H NMR spectra with a fixed total concentration 10 mM of (a) complex **4** (b) ligand **1** and complex **4** in a 1:1 ratio (c) ligand **1** in DMSO-d6 at 25 °C

**
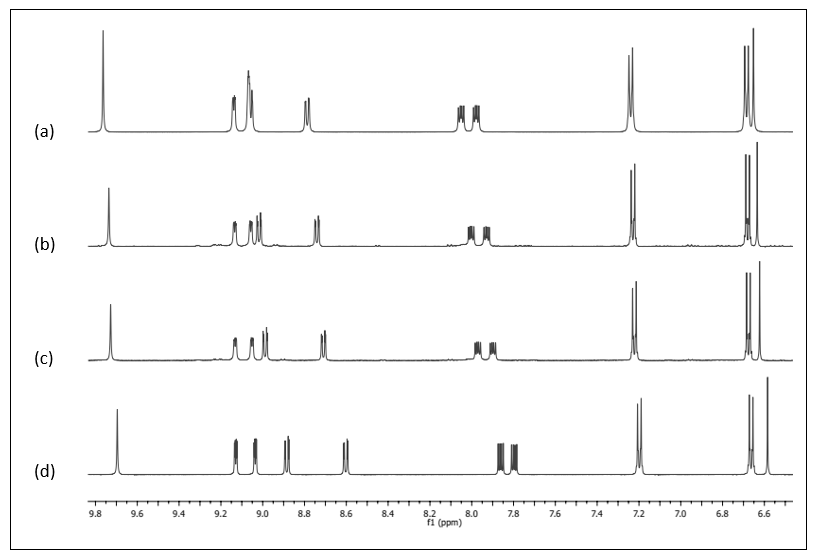
**

**Figure S28**: ^1^H NMR spectra with a fixed total concentration 10 mM of (a) complex **5** (b) ligand **2** and complex **5** in a 1:1 ratio (c) ligand **2** and complex **5** in a 2:1 ratio (d) ligand **2** in DMSO-d6 at 25 °C.


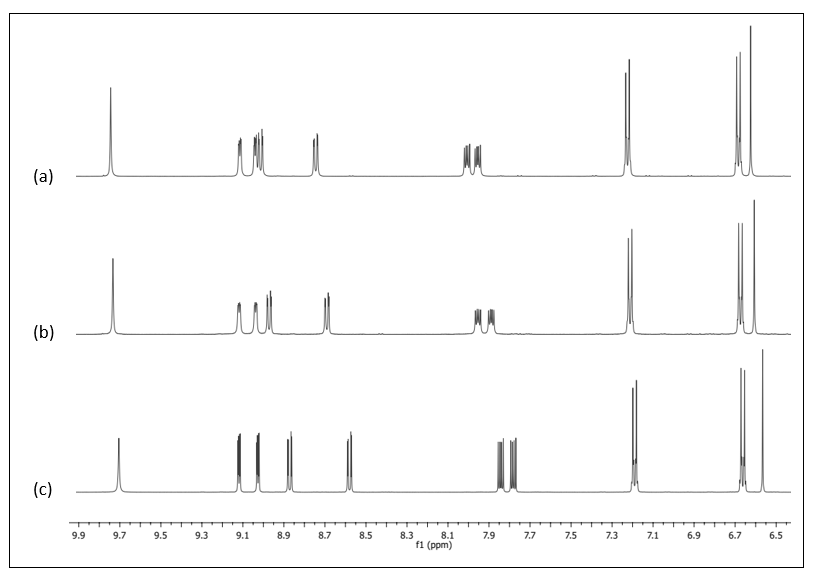


**Figure S29**: ^1^H NMR spectra with a fixed total concentration 10 mM of (a) complex **6** (b) ligand **3** and complex **6** in a 1:1 ratio (c) ligand **3** in DMSO-d6 at 25 °C.

**Section 6 - Results of Biological Testing**

**Table S2:** *In vitro* inhibitory effects of test samples determined as their ability after 24 h incubation to limit the growth of *C. albicans* in minimal media*

| Test sample and dosage | | | | Test sample and dosage | | | |
| --- | --- | --- | --- | --- | --- | --- | --- |
| Ligand | 60 μM | 30 μM | 15 μM | Silver Salt | 60 μM | 30 μM | 15 μM |
|  | Percentage Growth^#^ | | |  | Percentage Growth^#^ | | |
| **1** | 67% | 99% | 100% | **4** | 15% | 12% | 11% |
| **2** | 17% | 80% | 100% | **5** | 12% | 10% | 9% |
| **3** | 18% | 27% | 57% | **6** | 11% | 12% | 14% |
|  |  |  |  | AgClO_4_ | 9% | 10% | 12% |

*When the same experiments were conducted in nutrient-rich YEPD media no inhibition was observed with any test compound across the dosage range studied, 60-15 μM.

^#^Percentage Growth is measured relative to a control sample where the *C. albicans* was grown in minimal media where 100% = no inhibition and 0% = complete inhibition of growth. Potential impact of residual organic solvent is ruled out by the observation that growth in 2.5% v/v DMSO in media is greater than 96% of that of the control. Values stated are the average of triplicate measurements (n=3)

**Table S3**: *In vitro* inhibitory effects of test samples determined as their ability after 24 h incubation to limit the growth of *C. albicans* in YEPD media following standing of test compounds in solution for time periods between 0-72 h.

- Row A = test compounds taken from freshly prepared solutions
- Rows B-F = test compounds taken from previously prepared solutions which had been standing in the dark at rt for the length of time indicated

Data shown for complex **4** – Identical results were obtained for all test compounds

|  | Time standing in media (h) prior to testing | Percentage Growth^#^  60 μM dose | Percentage Growth^#^  30 μM dose | Percentage Growth^#^  15 μM dose |
| --- | --- | --- | --- | --- |
| A | 0 | 100 | 100 | 100 |
| B | 3 | 100 | 100 | 100 |
| C | 6 | 100 | 100 | 100 |
| D | 24 | 100 | 100 | 100 |
| E | 48 | 100 | 100 | 100 |
| F | 72 | 100 | 100 | 100 |

^#^ Percentage growth is determined relative to a control sample where 100% growth = no inhibition, 0% = complete inhibition of growth. Potential impact of residual organic solvent is ruled out by the observation that growth in 2.5% v/v DMSO in media is greater than 96% of that of the control. Values stated are the average of triplicate measurements (n=3)

**Table S4**: *In vitro* inhibitory effects of test samples determined as their ability after 3-48 h incubation to limit the growth of *C. albicans* in minimal media.

|  | Test Sample | | | | | | |
| --- | --- | --- | --- | --- | --- | --- | --- |
|  | Ligands | | | Silver(I) Salts | | | |
| Incubation Time | **1** | **2** | **3** | **4** | **5** | **6** | AgClO_4_ |
|  | Percentage Growth^#^ at 60 μM, 30 μM and 15 μM dosage levels | | | | | | |
| 3 h | 90, 93,95% | 93, 93, 94% | 100, 100, 100% | 100, 100, 97% | 100, 100, 100% | 100, 100, 100% | 100, 90, 93% |
| 6 h | 75, 83, 91% | 75, 78, 84% | 100, 92, 87% | 84, 75, 74% | 95, 81, 75% | 87, 79, 79% | 100, 77, 79% |
| 24 h | 68, 99, 100% | 17, 80, 100% | 18, 27, 57% | 15, 12, 11% | 12, 10, 9% | 11, 12, 14% | 9, 10, 12% |
| 48 h | 100, 100, 100% | 64, 100, 100% | 24, 45, 95% | 19, 19, 17% | 13, 10, 9% | 15, 17, 21% | 11, 91, 100% |

^#^ Percentage growth is determined relative to a control sample where 100% growth = no inhibition, 0% = complete inhibition of growth. Potential impact of residual organic solvent is ruled out by the observation that growth in 2.5% v/v DMSO in media is greater than 96% of that of the control. Values stated are the average of triplicate measurements (n=3)

**Tables S5-S11:** *In vitro* inhibitory effects of test samples determined as their ability after 24 h incubation to limit the growth of *C. albicans*, following prior standing of test solution in minimal media for time periods between 0-72 h.

- Row A = test compounds taken from freshly prepared solutions
- Rows B-F = test compounds taken from previously prepared solutions which had been standing in the dark at rt for the length of time indicated

**Table S5** – Inhibitory effect of ligand **1**.

|  | Time standing in minimal media (h) | Percentage Growth^#^  60 μM dose | Percentage Growth^#^  30 μM dose | Percentage Growth^#^  15 μM dose |
| --- | --- | --- | --- | --- |
| A | 0 | 67 | 99 | 100 |
| B | 3 | 55 | 95 | 100 |
| C | 6 | 54 | 93 | 97 |
| D | 24 | 80 | 100 | 100 |
| E | 48 | 59 | 95 | 99 |
| F | 72 | 69 | 100 | 100 |

^#^ Percentage growth is determined relative to a control sample where 100% growth = no inhibition, 0% = complete inhibition of growth. Potential impact of residual organic solvent is ruled out by the observation that growth in 2.5% v/v DMSO in media is greater than 96% of that of the control. Values stated are the average of triplicate measurements (n=3).

**Table S6:** Inhibitory effect of ligand **2**.

|  | Time standing in minimal media (h) | Percentage Growth^#^  60 μM dose | Percentage Growth^#^  30 μM dose | Percentage Growth^#^  15 μM dose |
| --- | --- | --- | --- | --- |
| A | 0 | 17 | 80 | 100 |
| B | 3 | 16 | 72 | 100 |
| C | 6 | 13 | 73 | 97 |
| D | 24 | 20 | 99 | 100 |
| E | 48 | 14 | 76 | 96 |
| F | 72 | 18 | 90 | 98 |

^#^ Percentage growth is determined relative to a control sample where 100% growth = no inhibition, 0% = complete inhibition of growth. Potential impact of residual organic solvent is ruled out by the observation that growth in 2.5% v/v DMSO in media is greater than 96% that of the control. Values stated are the average of triplicate measurements (n=3).

**Table S7:** Inhibitory effect of ligand **3**.

|  | Time standing in minimal media (h) | Percentage Growth^#^ 60 μM dose | Percentage Growth^#^ 30 μM dose | Percentage Growth^#^ 15 μM dose |
| --- | --- | --- | --- | --- |
| A | 0 | 18 | 27 | 59 |
| B | 3 | 24 | 36 | 65 |
| C | 6 | 19 | 17 | 31 |
| D | 24 | 20 | 61 | 94 |
| E | 48 | 21 | 39 | 88 |
| F | 72 | 15 | 37 | 69 |

^#^ Percentage growth is determined relative to a control sample where 100% growth = no inhibition, 0% = complete inhibition of growth. Potential impact of residual organic solvent is ruled out by the observation that growth in 2.5% v/v DMSO in media is greater than 96% of that of the control. Values stated are the average of triplicate measurements (n=3).

**Table S8:** Inhibitory effect of AgClO_4_.

|  | Time standing in minimal media (h) | Percentage Growth^#^ 60 μM dose | Percentage Growth^#^ 30 μM dose | Percentage Growth^#^ 15 μM dose |
| --- | --- | --- | --- | --- |
| A | 0 | 17 | 80 | 100 |
| B | 3 | 16 | 72 | 100 |
| C | 6 | 13 | 73 | 97 |
| D | 24 | 20 | 99 | 100 |
| E | 48 | 14 | 76 | 96 |
| F | 72 | 18 | 90 | 98 |

^#^ Percentage growth is determined relative to a control sample where 100% growth = no inhibition, 0% = complete inhibition of growth. Potential impact of residual organic solvent is ruled out by the observation that growth in 2.5% v/v DMSO in media is greater than 96% of that of the control. Values stated are the average of triplicate measurements (n=3).

**Table S9:** Inhibitory effect of complex **4**.

|  | Time standing in minimal media (h) | Percentage Growth^#^ 60 μM dose | Percentage Growth^#^ 30 μM dose | Percentage Growth^#^ 15 μM dose |
| --- | --- | --- | --- | --- |
| A | 0 | 15 | 12 | 11 |
| B | 3 | 13 | 13 | 12 |
| C | 6 | a11 | 11 | 12 |
| D | 24 | 13 | 13 | 13 |
| E | 48 | 15 | 17 | 18 |
| F | 72 | 12 | 12 | 14 |

^#^ Percentage growth is determined relative to a control sample where 100% growth = no inhibition, 0% = complete inhibition of growth. Potential impact of residual organic solvent is ruled out by the observation that growth in 2.5% v/v DMSO in media is greater than 96% of that of the control. Values stated are the average of triplicate measurements (n=3).

**Table S10:** Inhibitory effect of complex **5**.

|  | Time standing in minimal media (h) | Percentage Growth^#^ 60 μM dose | Percentage Growth^#^ 30 μM dose | Percentage Growth^#^ 15 μM dose |
| --- | --- | --- | --- | --- |
| A | 0 | 12 | 10 | 9 |
| B | 3 | 10 | 9 | 8 |
| C | 6 | 9 | 8 | 8 |
| D | 24 | 12 | 11 | 10 |
| E | 48 | 11 | 10 | 9 |
| F | 72 | 10 | 8 | 7 |

^#^ Percentage growth is determined relative to a control sample where 100% growth = no inhibition, 0% = complete inhibition of growth. Potential impact of residual organic solvent is ruled out by the observation that growth in 2.5% v/v DMSO in media is greater than 96% of that of the control. Values stated are the average of triplicate measurements (n=3).

**Table S11:** Inhibitory effect of complex **6**.

|  | Time standing in minimal media (h) | Percentage Growth^#^ 60 μM dose | Percentage Growth^#^ 30 μM dose | Percentage Growth^#^ 15 μM dose |
| --- | --- | --- | --- | --- |
| A | 0 | 11 | 12 | 14 |
| B | 3 | 13 | 11 | 13 |
| C | 6 | 11 | 9 | 9 |
| D | 24 | 14 | 12 | 19 |
| E | 48 | 25 | 20 | 19 |
| F | 72 | 12 | 10 | 13 |

^#^ Percentage growth is determined relative to a control sample where 100% growth = no inhibition, 0% = complete inhibition of growth. Potential impact of residual organic solvent is ruled out by the observation that growth in 2.5% v/v DMSO in media is greater than 96% of that of the control. Values stated are the average of triplicate measurements (n=3).
